# Excitonically Coupled Simple Coacervates via Liquid/Liquid Phase Separation

**DOI:** 10.1021/acs.jpclett.2c02466

**Published:** 2022-10-28

**Authors:** Anna R. Johnston, Gregory M. Pitch, Eris D. Minckler, Ivette G. Mora, Vitor H. Balasco Serrão, Eric A. Dailing, Alexander L. Ayzner

**Affiliations:** † Department of Chemistry and Biochemistry, University of California—Santa Cruz, Santa Cruz, California95064, United States; ‡ Biomolecular cryo-Electron Microscopy Facility, University of California—Santa Cruz, Santa Cruz, California95064, United States; § The Molecular Foundry, Lawrence Berkeley National Laboratory, Berkeley, California94720, United States

## Abstract

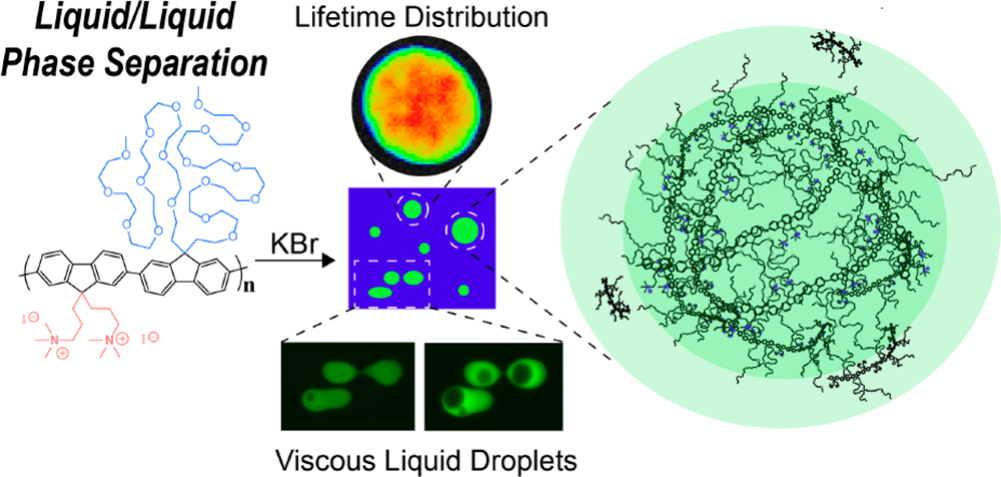

Viscoelastic liquid coacervate phases that are highly enriched in nonconjugated polyelectrolytes are currently the subject of highly active research from biological and soft-materials perspectives. However, formation of a liquid, electronically active coacervate has proved highly elusive, since extended π-electron interactions strongly favor the solid state. Herein we show that a conjugated polyelectrolyte can be rationally designed to undergo aqueous liquid/liquid phase separation to form a liquid coacervate phase. This result is significant both because it adds to the fundamental understanding of liquid/liquid phase separation but also because it opens intriguing applications in light harvesting and beyond. We find that the semiconducting coacervate is intrinsically excitonically coupled, allowing for long-range exciton diffusion in a strongly correlated, fluctuating environment. The emergent excitonic states are comprised of both excimers and H-aggregates.

Spontaneous separation of aqueous polyelectrolyte solutions into dilute and highly concentrated liquids is a fascinating process relevant to understanding the formation of early membrane-less organelles.^1,2^ Liquid/liquid phase separation has multiple exciting applications, which stem from the attractive properties of the concentrated viscoelastic fluid phase. This highly polymer-enriched aqueous *liquid* phase, called a coacervate, is of interest for drug design, catalysis, biomaterials, and underwater adhesives.^3−6^ Yet to date, the polyelectrolyte components of such coacervate phases have been electronically inactive.^7−14^ The formation of a liquid semiconducting coacervate would open exciting new application possibilities in light-harvesting and electronically conducting soft matter. In such a crowded aqueous system, inter- and intrachain electronic couplings between semiconducting polymer chains would support long-range exciton and charge motion. The strong fluctuations associated with a liquid state would couple to the electronic states of the system, both by influencing the ensemble of chain conformations and by direct interactions between small ions and extended π-electron states. Thus, a local trap state for an exciton in one instance may no longer be a trap in the next. At the same time, the liquid environment would allow for molecular diffusion. Such a combination is attractive from a photosynthetic perspective. We envision that a semiconducting coacervate droplet could in principle be encapsulated in a larger assembly and thereby serve as a photoactive compartment within an overarching soft artificial photosystem.

Formation of a semiconducting coacervate is also quite intriguing from fundamental considerations. The role of *extended* π-electron interactions on the thermodynamics of liquid/liquid phase separation, as well as the influence of the coupling between ionic and electronic degrees of freedom on coacervate photophysics, are highly underexplored.^14−17^ We expect the semiconducting coacervate to exhibit strongly correlated many-body interactions, the elucidation of which is likely to lead to the formation of novel electronic soft materials.

Typically, aqueous phase separation of electronically active conjugated polyelectrolytes (CPEs) leads to precipitants, colloidal gels, and complex fluids in which a solid-like phase persists.^15−18^ To the best of our knowledge there are no examples of true semiconducting *liquid* coacervates. We hypothesized that an alternating copolymer CPE composed of one ionic monomer and one highly polar nonionic monomer would have an increased probability of stabilizing a liquid coacervate phase. We reasoned that, in the limit where long-range electrostatic interactions are strongly screened, enhanced local dipolar interactions of nonionic side chains with solvent molecules and small ions would compete with interchain π-stacking. The latter strongly favors the formation of solid phases. Thus, we synthesized a novel polyfluorene-based CPE bearing ionically charged side chains on one monomer and oligo(ethylene glycol) (oEG) side chains with a substantial number of repeat units on the other (PFNG9, Scheme 1, Section S1 of the Supporting Information). We find that in the presence of high-ionic-strength potassium bromide (KBr) solutions, PFNG9 undergoes true liquid/liquid phase separation and forms spherical coacervate droplets with photophysical properties that differ substantially from the surrounding dilute solution. To the best of our knowledge, this is the first example of a semiconducting liquid coacervate.

**Scheme 1:**
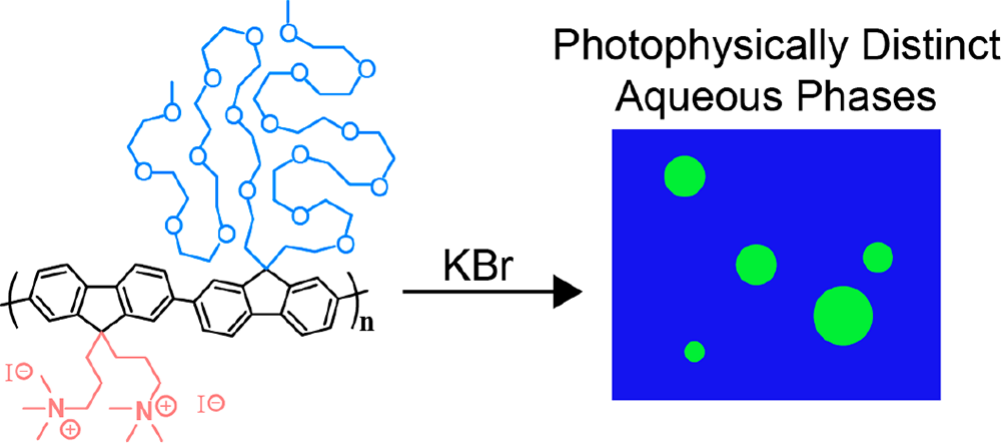
Conjugated Polyelectrolyte PFNG9 Undergoes Spontaneous Liquid/Liquid Phase Separation in High-Ionic-Strength Aqueous KBr Solutions

Figure 1A shows the wide-field differential interference contrast (TL-DIC) light microscopy image of an aqueous sample that contains PFNG9 (4.6 mg/mL; 2.8 mM in monomer) and 5.0 M KBr. Spherical liquid droplets are seen to be dispersed through the background dilute phase—an appearance that differs drastically from all other reported CPE-based complex fluids.^15−18^Figure 1B shows the corresponding photoluminescence (PL) image where the sample was excited between 340 and 380 nm and emission was collected between 450 and 490 nm. Within these illumination and emission bands, the dilute-phase PL is strongly enhanced, while droplets appear significantly darker. In contrast, when illuminating the sample between 450 and 490 nm and collecting emission between 500 and 550 nm (Figure 1C), we find that the dilute phase is darkened while the coacervate droplets are highly fluorescent. Clearly, the two phases are photophysically distinct from one another and the dissolved CPE in the absence of KBr (Figure S13)

**Figure 1:**
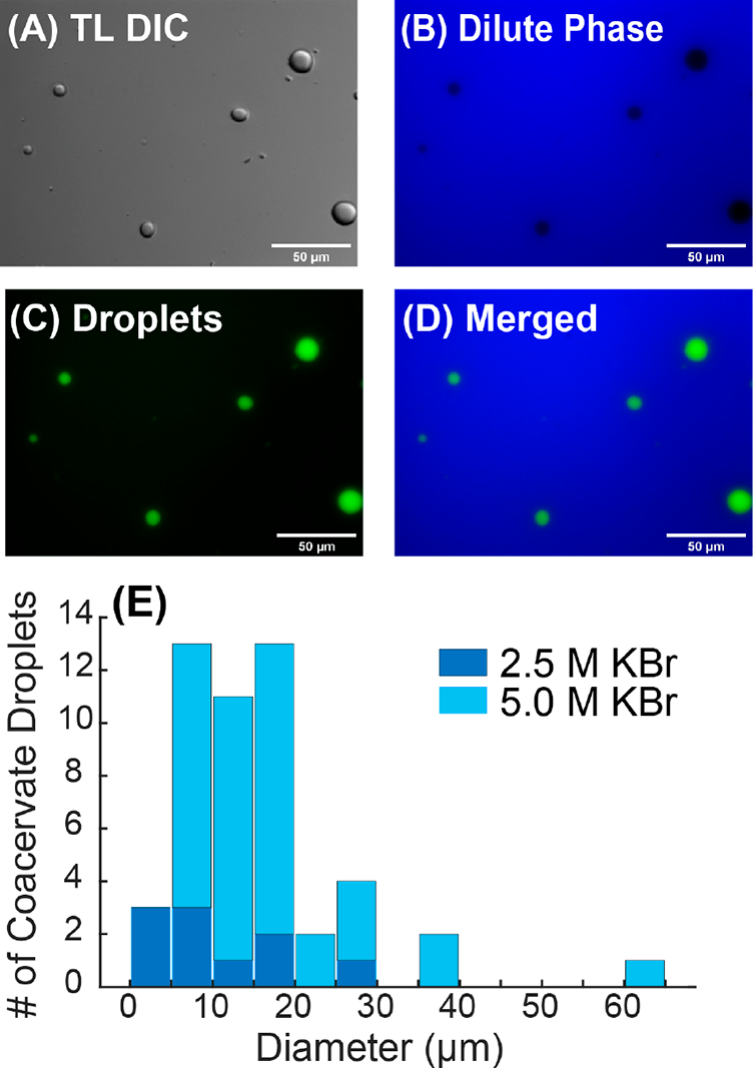
Images of CPE-based coacervate droplets. (A) TL-DIC image of the phase coexistence. (B) PL image exciting between 340 and 380 and collecting emission between 450 and 490 nm. (C) PL image exciting between 450 and 490 nm and collecting emission between 500 and 550 nm. (D) Merged PL image. (E) Comparison of the number of coacervate droplets vs droplet diameter between 2.5 and 5.0 M KBr samples. Distribution was collected using 5 images at each salt concentration.

We observed a phase transition from more precipitant-like, fractal particle morphologies to the characteristic liquid droplet morphology conventionally associated with coacervates. Figure S14 of the Supporting Information shows that this transition occurs between 2.5 and 5.0 M KBr with the disappearance of fractal particles at 2.5 M KBr, giving way to well-defined droplets at 5.0 M KBr (Figure S14C,F). We quantified the droplet size distribution using light microscopy at 2.5 and 5.0 M KBr, which is shown in Figure 1E. The number density and the observed size range of coacervate droplets are substantially larger at 5.0 M KBr. It is important to underscore that this distribution reflects droplets that could be imaged using optical microscopy. Droplets that are smaller than the diffraction limit would not be counted. In fact, we find that there are many such nanoscale and mesoscale droplets, as seen in cryogenic-transmission electron microscopy (cryo-TEM) images (Figure S24 of the Supporting Information). Thus, the calculated size distributions from optical microscopy primarily reflect the micrometer-scale subpopulation.

Figure 2 shows the time points of a PL microscopy video in which the flow behavior of the droplets can be observed. The full video is provided as Supporting Information. We find that the dynamics are quite slow, which is consistent with the high apparent viscosity of the concentrated phase observed when handling the sample. The droplet dynamics are characteristic of a true viscous liquid as opposed to a colloidal gel.^14−17^

**Figure 2:**
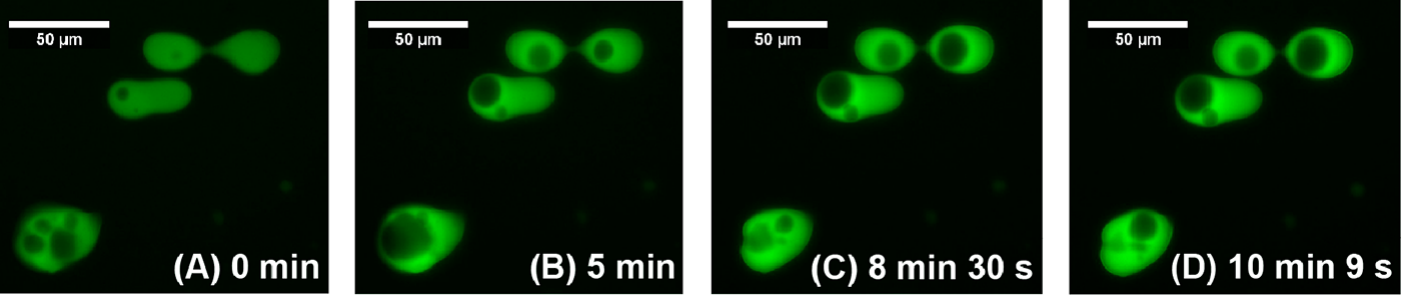
PFNG9 coacervate droplets in the presence of 5.0 M KBr imaged over 10 min and 9 s. The full video is available in the Supporting Information.

One of the most interesting aspects of a semiconducting coacervate is the influence of the highly correlated and strongly fluctuating environment on the ensemble of electronic states of the constituent CPE chains. To interrogate the emergent photophysical properties associated with the formation of this coacervate phase, we used a combination of steady-state and time-resolved PL spectroscopy methods. Figure 3A shows absorption or optical density (OD) spectra of dilute and concentrated phases, which were acquired by carefully separating the phases. Upon the addition of 5.0 M KBr, the OD spectrum of the dilute phase undergoes a mild redshift relative to aqueous PFNG9 solutions without added salt. This likely reflects an increased propensity for intrachain π-stacking interactions as repulsion between ionic side chains becomes strongly screened. In contrast to the mild redshift for the dilute solution, the OD spectrum of coacervate droplets acquires a substantial red shoulder, which implies that new electronic states form within the coacervate.

**Figure 3:**
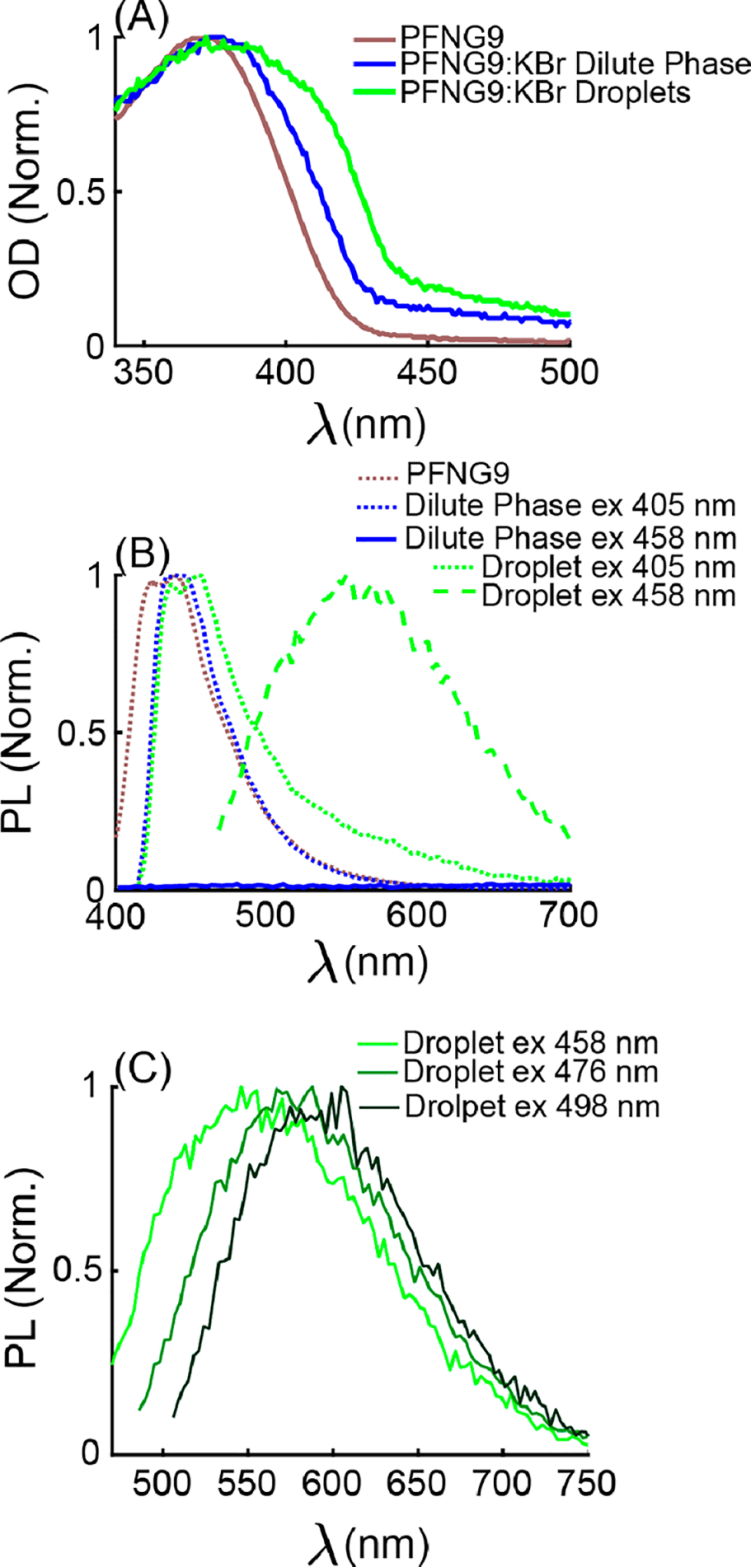
(A) Optical density (OD) and (B) photoluminescence (PL) spectra of PFNG9 compared to the separated phases of PFNG9 with 5.0 M KBr. (C) No emission is shown from the dilute phase upon excitation at 458 nm and an excitation wavelength dependence to the droplet emission. PL spectra shown in (B) and (C) were collected using xyλ-scan confocal microscopy and by defining regions of interest containing dilute solution or droplets from which to measure the PL signal.

To directly compare PL spectra of the dilute solution and the coacervate droplets, we used laser-scanning confocal microscopy. Figure 3B shows that, when exciting at 405 nm, the dilute solution and the coacervate display a blue emission band that decays by ∼550 nm. At the same excitation wavelength, the coacervate phase gives an enhanced PL intensity on the red side of the dilute-solution PL spectrum, consistent with wide-field PL microscopy images in Figure 1. Intriguingly, when exciting at 458 nm near the onset of red shoulder in the coacervate OD spectrum, the droplets exhibit a new, broad green emission band. We observed a similarly broad green band for the concentrated phase when physically separating the concentrated phase from the dilute phase and performing bulk PL measurements (Figure S21B).

The appearance of this green band (commonly shortened to g-band) has previously been observed in a number of polyfluorene derivatives.^19,20−40^ However, its nature continues to be controversial.^18,19,24,25,30,32−37^ Initial reports suggested that it was due to the formation of excimers,^25,26,33,34,41,42^ implying the presence of interchromophore interactions in the excited state. On the basis of early single-molecule spectroscopy measurements on (nonionic) polyfluorenes and measurements on random fluorene-*co*-fluorenone copolymers, others have argued that the g-band is entirely due to fluorenone defects on *single* chains.^39,40^ However, recent measurements from more comprehensive single-molecule studies,^37,38^ as well as from the controlled synthesis of fluorene and fluorenone oligomers,^27^ have cast serious doubt on the hypothesis that fluorenone defects on isolated polymer chains are solely responsible for the g-band. The totality of the recent work suggests that the g-band may be composed of H-aggregate exciton states as well as fluorenone-defect-based states.

We stress that PFNG9 chains in the dilute solution surrounding coacervate droplets display no g-band emission. In dilute solution, PFNG9 chains are effectively isolated. Therefore, we conclude that the g-band emission *cannot be explained by fluorenone defects on single chains*. To further probe the nature of the PFNG9 g-band within the coacervate, we went on to measure the recovery of the PL signal after light exposure to elucidate whether the coacervate was undergoing an irreversible photochemical reaction. We found the PL intensity for both the dilute solution and droplets fully recovered after ∼1 and 30 s light exposure under the microscope (Figures S18 and S19). These results do not support the hypothesis that photodegradation by irreversible formation of fluorenone defects in the CPE backbone is leading to g-band emission. Although our results do not preclude the possibility of *reversible* fluorenone formation,^39^ we note that the bulk solution was degassed with Ar(g) prior to measurements, and during image collection the coverslip was sealed with Kapton tape to minimize inward diffusion of oxygen.

What, then, is the physical origin of the g-band within the coacervate? We believe that a strong hint is provided by the evolution of the droplet PL spectrum with increasing excitation wavelength, which was collected using confocal microscopy, shown in Figure 3C. It is often the case that emission spectra of conjugated polymers in the solid state are independent of the excitation wavelength over wide regions of the absorption spectrum. In contrast, the data in Figure 3C show that the structure of the g-band undergoes significant changes: The shoulder on the blue side of the spectrum disappears as the excitation wavelength goes from 458 to 498 nm. This suggests that different populations of distinct emitting species are excited as the excitation wavelength is increased. The presence of two emissive species is supported by the approximate decomposition of the PL spectra in Figure 3C into two distinct contributions with excitation wavelength-dependent amplitudes, shown in Figure S23 of the Supporting Information. Our results are consistent with single-molecule measurements by Nakamura et al., which provided evidence that the g-band of polyfluorene chains with *intrachain* interactions consisted of multiple emitting species.^37^

Given the inherent proximity between chains within the crowded coacervate environment and the lack of g-band emission in the dilute phase, we argue that the coacervate g-band is likely primarily composed of *interchain* exciton states.^43,44^ The fact that only one new, relatively narrow absorption band appears within the coacervate but that two putative emissive species comprise the g-band PL spectrum is consistent with a coexistence of excimers^44^ and H-aggregate excitons within a coacervate droplet. Evidence for H-aggregate formation is provided by the appearance of a new red-shifted absorption band in the OD spectrum of the coacervate compared to the dilute phase (Figure 1A). In contrast, excimers result from interchromophore interactions in the excited state only and thus do not give rise to new absorption bands. Excimer states are characterized by broad and unstructured emission spectra, as the ground-state energy as a function of interchromophore separation for an excimer configuration does not correspond to a bound state.^36,41^ Although the ground state of an H-aggregate is bound, unlike in an ordered thin film, in viscous droplets we expect a broad range of excitonic coupling strengths. The result is a relatively broad ensemble H-aggregate spectrum where the vibronic structure is likely largely washed out. Thus, distinguishing excimers and H-aggregates based on the widths, shapes, and peak positions of their ensemble PL spectra within the coacervate is not straightforward.^45^

We note that our conclusion does not preclude the possibility that fluorenone defects also contribute to the coacervate emission spectrum. However, it must still then be the case that emission from fluorenone-based states *requires an interchromophore excitonic coupling*.^32^ Thus, we conclude that the CPE coacervate is an intrinsically excitonically coupled viscoelastic liquid. This is summarized in a schematic in Scheme 2.^46,47^

**Scheme 2:**
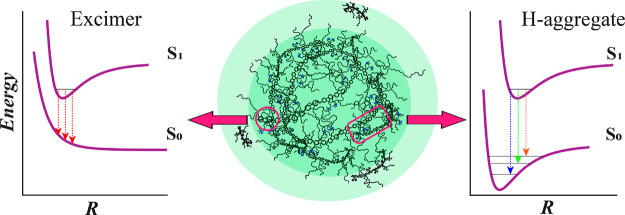
Schematic of a PFNG9 Coacervate Droplet (middle) and Illustrations of the Corresponding Potential Energy Curves as a Function of the (Average) Interchromophore Separation *R* Shown in the Side Panels Regions corresponding to excimer and H-aggregate exciton states are speculatively labeled as magenta domains with few or extended interchain contacts, respectively.^44,45^. In practice, the potential energy surfaces are likely complex landscapes with multiple minima.

To gain a better understanding of the coacervate photophysics, we used fluorescence lifetime imaging (FLIM) to characterize the radiative relaxation of dilute-phase and coacervate excitons. FLIM allows us to measure PL lifetimes as a function of position within the droplet. Figure 4A shows the heat map of PL lifetimes of a representative droplet following excitation at 445 nm while collecting emission in the 590 ± 25 nm region. The average PL lifetime  calculated for individual coacervate droplets was found to range between 560 and 830 ps. The PL lifetime histogram (Figure 4B) highlights differences in lifetime found throughout the droplet where the color coding of the histogram matches that in the FLIM heat map. Similar images and histograms were collected for 9 additional droplets (Figure S27).

**Figure 4:**
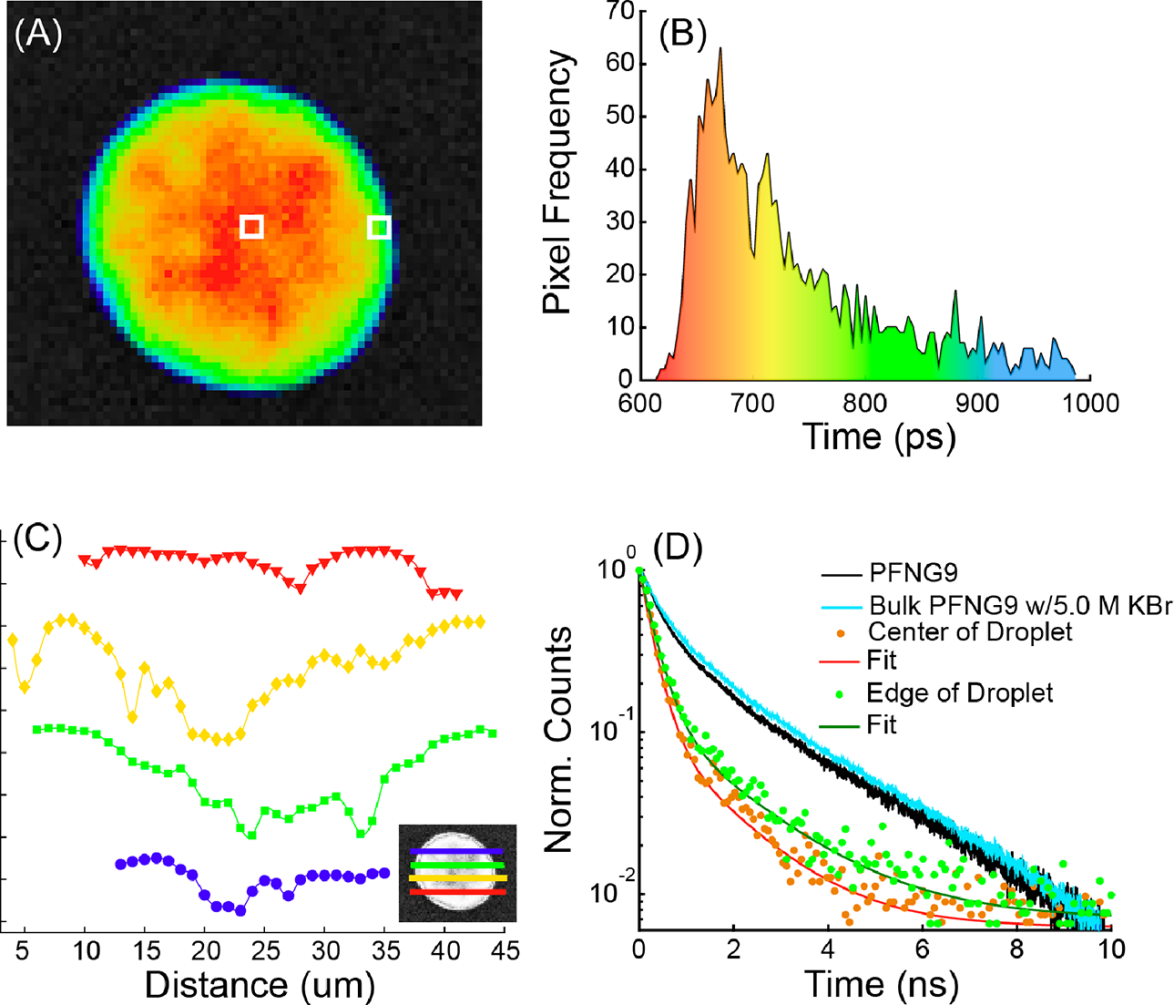
(A) Select droplet from a FLIM image, where pixel selection in the center and on the edge of the droplet are highlighted with white boxes. The grayscale image shows line cuts from which distant dependent lifetime fluctuations were pulled (see panel (C)). Excitation: 375 nm for the dilute phase, 445 nm for the coacervate. (B) Histogram of PL lifetimes measured across the entire droplet. (C) Distance dependent fluctuations in lifetime (symbols) taken from the line cuts shown in the grayscale image in (A), along with corresponding cubic spline curves (solid). Curves have been vertically offset for clarity. (D) PL decay curves and fits associated with the selected pixels in (A). Black and light blue decays are bulk-solution TRPL. Because the quantity of the dilute phase far exceeds that of the droplets, the bulk TRPL data is largely representative of the dilute phase , which was found to be 970 ps. The excitation/emission wavelengths are the same as in (A).

The FLIM heat map shows that  is a function of position within the droplet, demonstrating that  is a fluctuating variable within the coacervate. This observation is consistent with the viscous liquid macrostate. To characterize the approximate length scale of  fluctuations, in Figure 4C we plot 4 linecuts through the droplet shown in Figure 4A. The extracted image gray value as a function of position for the different linecuts shows that relatively small fluctuations in  occur on the ∼1 μm scale, while larger fluctuations are also seen on the ∼10 μm scale. Differences in  must reflect differences in local structure. We speculate that the large viscosity of the droplet leads to a relatively slow interconversion between large, strongly interacting CPE networks and relatively loosely associated domains with fewer interchain interactions. However, within the droplet interior, the mean fluctuation in  is not dramatic, as seen from the histogram in Figure 4B, suggesting relatively subtle differences in structure as a function of position.

It was commonly the case that the PL lifetime was somewhat longer near the edge of the droplets than in the center. This is shown in Figure 4C, which compares decays collected in the middle of the droplet to those of the near-surface region (indicated by white squares in Figure 4A). In going from the bulk to the surface of the droplet, the lifetime of the short component increases from 255 to 298 ps, while the lifetime of the long component increases from 1367 to 1693 ps. The difference between the bulk and the surface lifetimes increases closer to the edge of the droplet, as seen from the lifetime histogram and the corresponding FLIM image. For the regions labeled with white squares, we can quantify the change in the (intensity-weighted) contribution that each component makes to the total decay, , according to , where  and  are the amplitude and lifetime of component , respectively.  decreases by ∼8% while  increases by ∼16% for the near-surface region relative to the middle. Since longer lifetimes are often associated with more extended chains, we speculate that the PFNG9 backbone undergoes a relative extension near the surface. This could allow polymer chains to maximize the number of oEG side chains capable of orienting approximately normal to the droplet/solution interface, thereby likely lowering the surface free energy.

It is important to ask why PFNG9 forms a liquid coacervate phase while the overwhelming majority of CPEs do not. Although it is reasonable to expect that the oEG side chains are implicated, it is not immediately clear what contribution(s) they make to the system free energy such that the liquid state becomes stabilized at high [KBr]. In our system, there is no interaction with an oppositely charged polyelectrolyte, as would occur in a complex coacervate. Therefore, in the *simple* PFNG9 coacervate it is likely that the interaction between the excess ions and the oEG side chain plays a role in inducing the formation of this viscoelastic liquid phase. It is known that K^+^ ions readily interact with crown ethers, which are chemically related to the oEG side chains.^48,49^ We aimed to elucidate whether the ionic strength alone determined coacervate formation independent of ion identity or whether the identity of the cation was an important factor. We prepared similar samples using comparable concentrations of lithium bromide (LiBr), tertraethylammonium bromide (TEAB), and calcium bromide (CaBr_2_). Liquid coacervate formation was not observed in the presence of any other salts chosen for this study (Figures S15–S17). We also investigated whether the choice of anion played an important role in the phase behavior. We chose to evaluate whether coacervate formation took place in the presence of 5 M KF, with the expectation that the fluoride anion was substantially different in size and charge density compared to the bromide anion. We found that coacervate droplets were formed with KF as with KBr (see Figure S28). We conclude that the specific K^+^–oEG interaction is likely involved in the stabilization of the coacervate phase. We reason that the large viscosity of the droplets is then a consequence of both interchromophore π-stacking and a large number of K^+^ ions interacting with a correspondingly large number of ethylene glycol groups. The separate enthalpic and entropic contributions to the underlying free energy, including the competition between ion desolvation and side chain interactions, remain obscured at the moment. Nevertheless, the coexistence of a dilute solution with the simple coacervate phase appears to correspond to a global free energy minimum at room temperature (Figure S29).

In summary, we have demonstrated that the chemical structure of a CPE can be rationally designed to undergo liquid/liquid phase separation to stabilize a semiconducting coacervate macrostate, which is of fundamental interest to coacervate physical chemistry. We find that oligo(ethylene glycol) side chains are critical to the simple coacervation process, which involves the interaction between ethylene glycol units with K^+^ ions. The CPE coacervate is comprised of intrinsically excitonically coupled chains with rich exciton dynamics in the presence of a fluctuating ionic environment. In addition to its fundamental significance, this observation is intriguing from an applications standpoint. The electronic connectivity within the concentrated liquid could be used to move excitons, electrons, or holes through space over distances that are large compared to the monomer size. The strong coupling between and electronic and ionic degrees of freedom can in principle be used to manipulate this quasiparticle migration. These characteristics are likely to be desirable for light harvesting, catalysis, or sensing. Finally, semiconducting coacervate droplets may in principle be encapsulated in larger soft-matter assemblies, leading to the potential for compartmentalization and a significant increase in light-harvesting complexity.

## Supporting Information Available

Monomer and polymer synthesis details, additional optical microscopy, steady-state spectroscopy, cryo-TEM images, time-resolved photoluminescence spectroscopy, and additional fluorescence lifetime imaging results (PDF)
Slow droplet dynamics where the top middle of the field of view shows the gradual thinning of a neck connecting two adjacent droplets (MP4)
Transparent Peer Review report available (PDF)
