# Supplementary material for: Excitonically Coupled Simple Coacervates via Liquid/Liquid Phase Separation
Source: J Phys Chem Lett. 2022 Oct 28;13(44):10275–81. doi: 10.1021/acs.jpclett.2c02466 (PMC9661528; doi:10.1021/acs.jpclett.2c02466)
Supplement: Supplementary file 1 — jz2c02466_si_001.pdf [file jz2c02466_si_001.pdf]

Supporting Information

Excitonically Coupled Simple Coacervates via Liquid/Liquid Phase Separation

Anna R. Johnston[1†], Gregory M. Pitch[1†], Eris D. Minckler[1], Ivette G. Mora[1], Vitor H. Balasco Serrão[1,2], Eric A. Dailing[3], Alexander L. Ayzner[1]*

[1] *Department of Chemistry and Biochemistry, University of California, Santa Cruz, Santa Cruz, California, 95064, USA*

[2] *Biomolecular cryo-Electron Microscopy Facility, University of California, Santa Cruz, Santa Cruz, California, 95064, USA*

[3] *The Molecular Foundry, Lawrence Berkeley National Laboratory, Berkeley, CA, 94720, USA*

[†] Authors made equal contributions.
* aayzner@ucsc.edu

S1. Synthetic Methods

Starting Materials and Characterization.

Reagents and materials were used as received from the following distributors. Specifically, 2,7-dibromofluorene and 4-toluenesulfonyl chloride were obtained from Oakwood Chemical. Bis(pinacolato)diboron >98% and 1,4-dioxane anhydrous 99.8% were obtained from Alfa Aesar. Nonaethylene glycol monomethyl ether >93.0% was obtained from TCI America Inc. Tetraethylammonium bromide 100% was obtained from Chem-Impex Int'l Inc. Palladium catalyst [1,1'- Bis(diphenylphosphino)ferrocene] dichloropalladium (II), dimethyl aminopropyl chloride hydrochloride 96%, sodium hydride 60% dispersed in mineral oil, and dimethylformamide anhydrous 99.8% were obtained from Sigma-Aldrich. Methyl iodide 99.5%, tetrahydrofuran HPLC grade, dichloromethane 99.5% were obtained from Spectrum Chemical. Sodium hydroxide, sodium hydroxide 50% w/w, potassium carbonate anhydrous 99.7%, triethylamine 99%, diethyl ether 99%, methanol 99.9%, acetone HPLC grade, chloroform 99.9%,

ethyl acetate 99.5%, hexanes 98.5%, and potassium acetate >99% were obtained from Fisher Chemical. Dimethyl sulfoxide 99.7% was obtained from Acros Organics. Chemglass pressure tubes were used for polymerization (48 mL - part number CG-1880-04), and quaternization (350 mL - part number CG-1880-12). $CDCl_3$ (D 99.8%) and D_2O (D 99.9%) were both purchased from Cambridge Isotope Laboratories. Nuclear magnetic resonance (*NMR*) spectra of the monomers were collected with a Bruker Avance III HD 4 channel 500 MHz NMR, and spectra of the polymers were collected with a Bruker Avance III HD 4 channel 800 MHz NMR with a cryoprobe.

Preparation of Reagents.

Reagent TG9

Synthesis of (1-(p-tosyl)-3,6,9,12,15,18,21,24,27- nonaoxooctacosane To a clean and dried 25 mL round bottom flask placed in an ice bath, a Teflon-coated stir bar, NaOH (0.80 g, 20.0 mmol), DI H_2O (4.0 mL, 222 mmol), nonaethylene glycol monomethyl ether (G9OH, 3.00 g, 7.0 mmol), and THF (8.0 mL, 98.6 mmol) were added and allowed to react for 30 minutes. Next, PTSC (2.40 g, 12.6 mmol) was added under an atmosphere of N_2(g) and reacted for 12 hours. Upon completion, the reaction contents were dumped into 16 mL of cold DI H_2O, followed by extraction of the product with DCM (4 x 10 mL). The organic layer was washed with DI H_2O (2 x 10 mL), brine (1 x 10 mL), and subsequently dried over Na_2SO_4. The anhydrous organic layer was decanted away from the drying agent and concentrated under reduced pressure to provide the product TG9 as a colorless oil (99% yield, 4.06 g).[2] **Figure S1**: TG9 ^{1}H NMR (500 MHz, $CDCl_3$): δ 7.80- 7.79 (d, 2H), 7.35- 7.33 (d, 2H), 4.16 (t, 2H), 3.68 (t, 2H), 3.65- 3.64 (m, 22H), 3.61 (m, 4H), 3.58 (s,

4H), 3.54 (s, 2H), 3.37 (s, 3H), 2.44 (s, 3H) **Figure S2**: TG9 ^{13}C NMR (500 MHz, $CDCl_3$): δ 144.9, 133.2, 130.0, 128.1, 72.1, 70.9, 70.8, 70.7, 70.7, 69.4, 68.8, 68.1, 59.2, 25.8, 21.8.

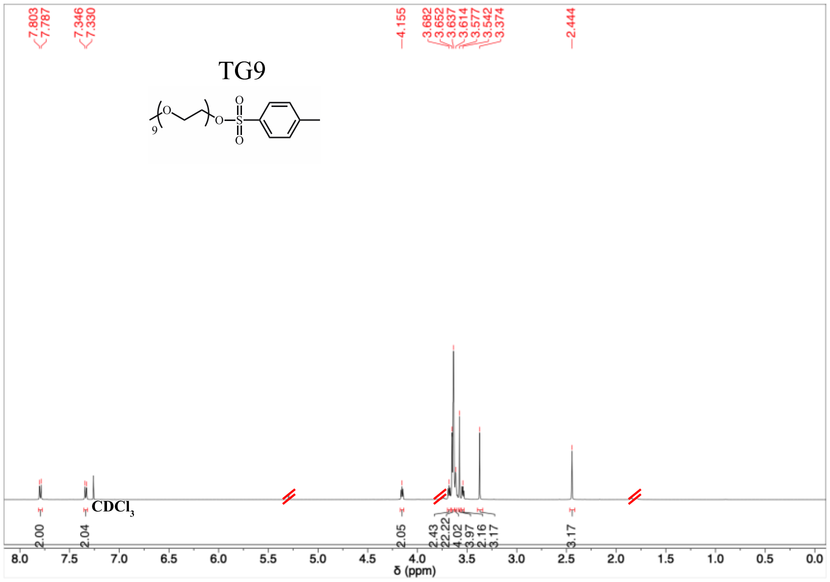

Figure S1: TG9 ^{1}H NMR with trace amount of *DCM*, *THF*, and water.

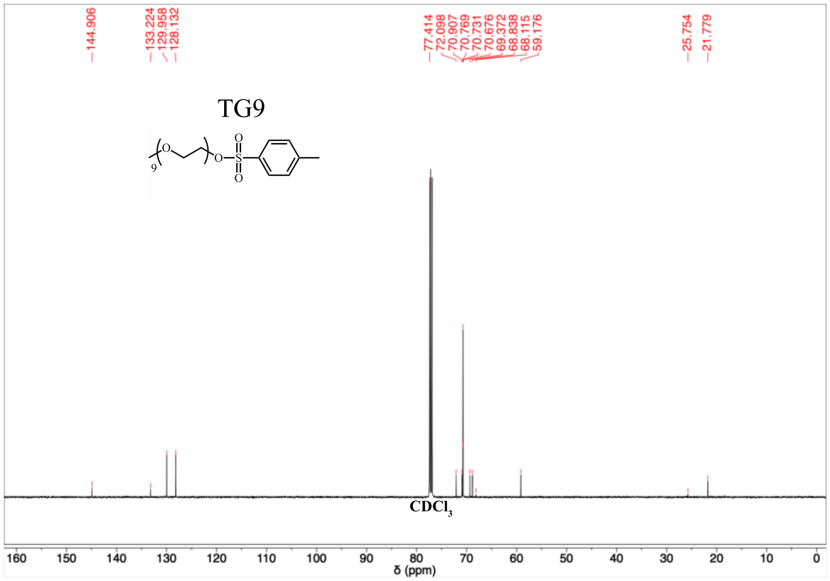

Figure S2: TG9 ^{13}C NMR

Synthesis of Monomers.

Monomer FN

Synthesis of (2,7-dibromo-9,9-bis(3'-(N,N-dimethyl-amino)-propyl)-fluorene) To a clean, dried, 100 mL two neck round bottom flask, a Teflon coated stir bar, dimethyl sulfoxide (DMSO, 30.9 mL, 434.6 mmol), 2,7- dibromofluorene (F, 2 g, 6.2 mmol), tetrabutylammonium bromide (TBAB, 39.8 mg, 0.12 mmol), and 4 mL of a 50 wt. % aqueous sodium hydroxide solution (50 wt. % aq. NaOH, 4 mL, 154.3 mmol) was added under an atmosphere of nitrogen (N_2(g)). An additional aliquot of DMSO (10.5 mL, 145.1 mmol) was added to the reaction flask, followed by dimethyl aminopropyl chloride hydrochloride salt (DAPCl, 2.6 g, 16.4 mmol). The reaction was stirred and heated at 60 °C for 12 hours. Reaction progress was monitored by thin-layer chromatography (TLC). Deionized water (DI H_2O, 40 mL, 2.216 mmol) was added to the reaction flask to dissolve precipitated salts as well as to solvate DMSO. The product (FNB) was extracted from the wet DMSO layer with diethyl ether (Et_2O, 8 x 25 mL), and washed with a 10 wt. % aqueous NaOH (10 wt. % aq. NaOH, 2 x 50 mL). The organic layer was washed with DI H_2O (3 x 50 mL), followed by a brine wash (1 x 50 mL), and then dried over anhydrous sodium sulfate (Na_2SO4). The concentration of the anhydrous organic layer under reduced pressure lead to crude solid which was purified with a silica gel column (Hexanes: Ethyl Acetate: Triethylamine, 49:49:2) to obtain FN (59% yield, 1.81 g).[4,5] **Figure S3**: FN ^{1}H NMR (500 MHz, $CDCl_3$): δ 7.52-7.50 (d,

2H), 7.47 (s, 2H), 7.45-7.44 (d, 2H), 2.03 (s, 12H), 1.99 (m, 8H), 0.77 (m, 4H) **Figure S4**: FN ^{13}C NMR (500 MHz, $CDCl_3$): δ 152.2, 139.3, 130.6, 126.3, 121.8, 121.4, 59.7, 55.5, 45.5, 37.7, 22.2.

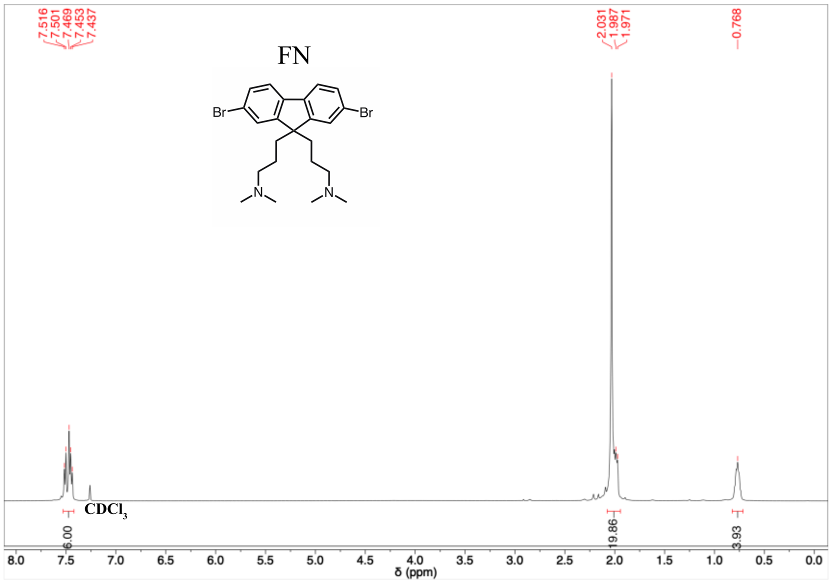

Figure S3: FN ^{1}H NMR

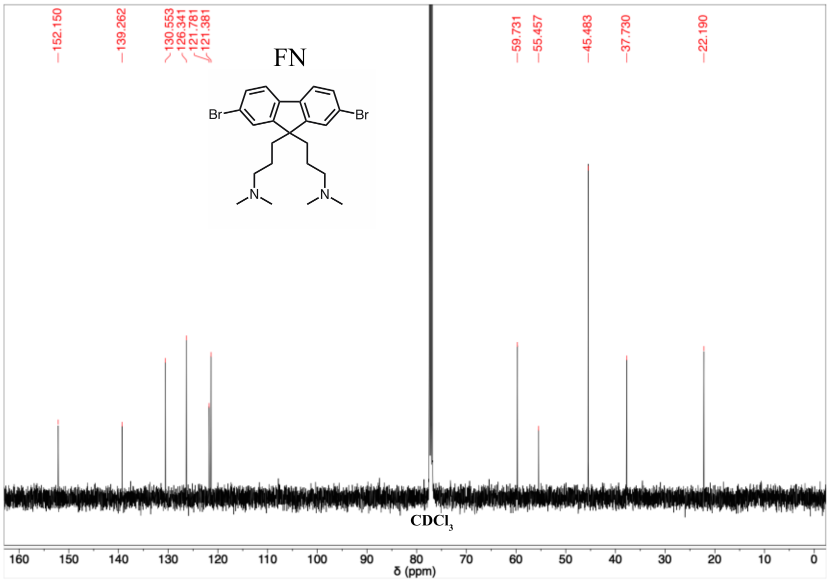

Figure S4: FN ^{13}C NMR

Monomer FNB

Synthesis of (2,7-diboryl pinacol ester-9,9-bis(3'-(N,N-dimethyl-amino)-propyl)-fluorene) To a clean, dried, 100 mL two neck round bottom flask, a Teflon coated stir bar, dimethylformamide (DMF, 39 mL, 505.8 mmol), FN (1 g, 2.0 mmol), bis(pinacolato)diboron (B_2Pin_2, 2.26 g, 8.9 mmol), potassium acetate (KOAc, 3.53 g, 17.8 mmol), [1,1'-Bis(diphenyl-phosphino)ferrocene]dichloro-palladium(II) (Pd(dppf)Cl_2, 0.296 g, 0.40 mmol) were added under an atmosphere of N_2(g). The contents of the reaction were stirred and heated at 80 °C for 24 hours. Reaction progress was monitored by TLC. Upon completion, the reaction was concentrated to dryness, and the crude solid was extracted with hot HPLC-grade hexanes (7 x 100 mL). The combined hexanes layer was filtered, concentrated to dryness, reextracted with hot hexanes, and re-concentrated to dryness. Acetone was used to extract the product from the re-dried hexanes layer and was allowed to crystallize out of the solution as an off-white solid. The FNB crystals were collected via filtration and washed with a minimal amount of cold acetone to obtain FNB (55% yield, 0.6647 mg).[4,5] **Figure S5**: FNB ^{1}H NMR (500 MHz, $CDCl_3$): δ 7.80-7.78 (d, 2H), 7.78 (s, 2H), 7.71-7.70 (d, 2H), 2.06 (m, 4H), 1.99 (m, 4H), 1.98 (s, 12H), 1.37 (s, 24H), 0.75 (m, 4H) **Figure S6**: FNB ^{13}C NMR (500 MHz, $CDCl_3$): δ 149.4, 144.0, 134.3, 129.1, 119.8, 84.0, 59.2, 54.7, 44.6, 37.4, 31.1, 25.1.

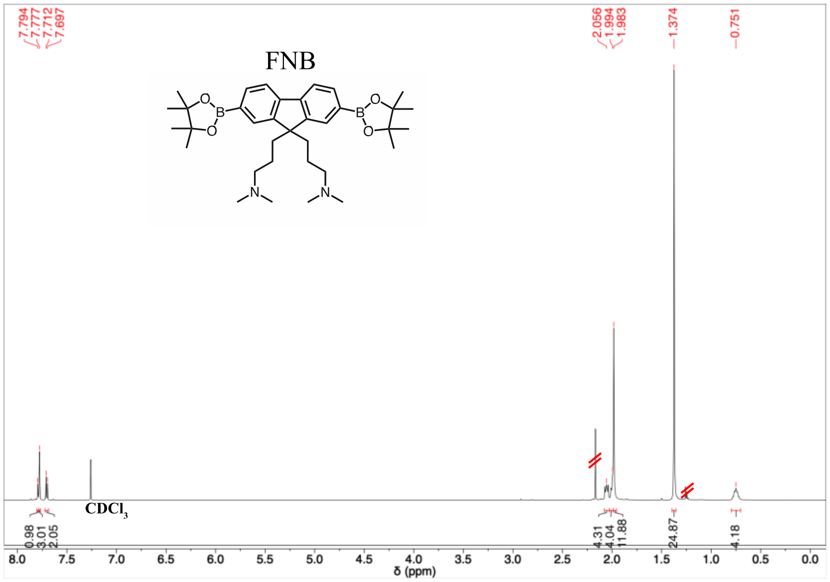

Figure S5: FNB ^{1}H NMR with trace amount of acetone and hexanes.

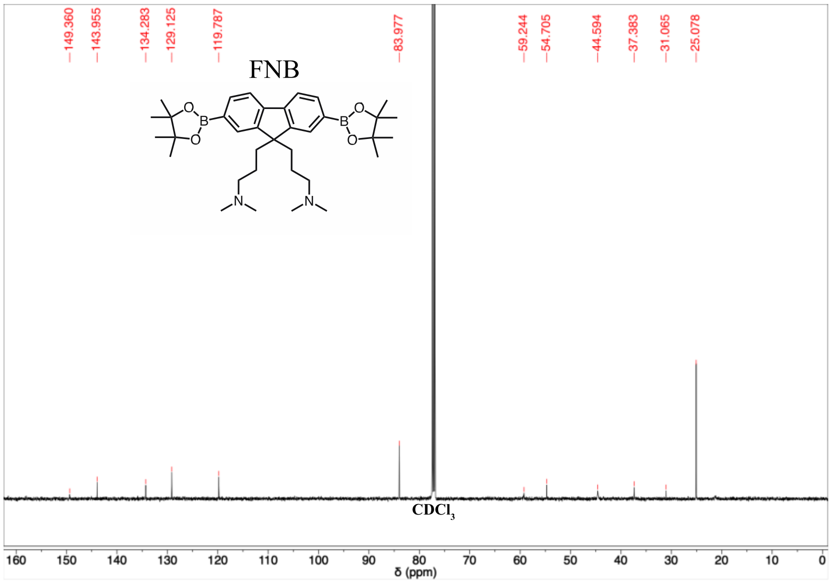

Figure S6: FNB ^{13}C NMR

Monomer FG9

Synthesis of (2,7-dibromo-9,9-bis-(2-(2-(2-(2-(2-(2-(2-(2-methoxy-ethoxy) ethoxy) ethoxy) ethoxy) ethoxy) ethoxy) ethoxy) ethoxy) ethyl)- fluorene) To a clean and dried 50 mL round bottom flask, a Teflon coated stir bar, F (670 mg, 2.1 mmol), anhydrous DMF (12 mL, 155 mmol), and NaH in 60% w/w dispersed in mineral oil (210 mg, 5.3 mmol) were added under an inert atmosphere of N_2(g). After 30 minutes, the bright red solution was allowed to react at 60 °C for 12 hours with previously prepared TG9 (3.0 g, 5.2 mmol). After quenching the remaining NaH with DI H_2O (15 mL), the reaction was extracted with DCM (4 x 50 mL). Dried the combined organic layer over Na_2SO_4. The anhydrous organic layer was decanted away from the drying agent. While stirring, a mixture consisting of 95% DCM with 5% Methanol (MeOH) was used to wash the product from the Na_2SO_4 slurry. The DCM: MeOH solution was decanted from the Na_2SO_4, and combined with the organic layer. The organic layer was concentrated under reduced pressure to provide a semi-crude solid which was purified further via a silica gel column (Ethyl Acetate: MeOH, 90:10). Since the percentage of MeOH in the solvent used to elute the aggregated fraction of FG9 was 10%, FG9 was dissolved in chloroform ($CHCl_3$) to help precipitate out the once dissolved silica gel. The $CHCl_3$ solution was then filtered to remove the precipitate, and concentrated under reduced pressure to obtain FG9 (30.0% yield, 0.710 g).[3] **Figure S7**: FG9 ^{1}H NMR (500 MHz, $CDCl_3$): δ 7.53 (d, 2H), 7.52- 7.50 (d, 2H), 7.47-7.46 (d, 2H), 3.64 (m, 44H), 3.58 (m, 4H), 3.54 (t, 8H), 3.37 (m, 10H), 3.18 (t, 4H), 2.77 (t, 4H), 2.33 (t, 4H) **Figure S8**: FG9 ^{13}C NMR (500 MHz, $CDCl_3$): δ 151.0, 138.6, 130.8, 126.8, 121.7, 121.4, 72.0, 70.7, 70.7, 70.6, 70.6, 70.5, 70.1, 66.9, 60.5, 59.1, 52.0, 39.6.

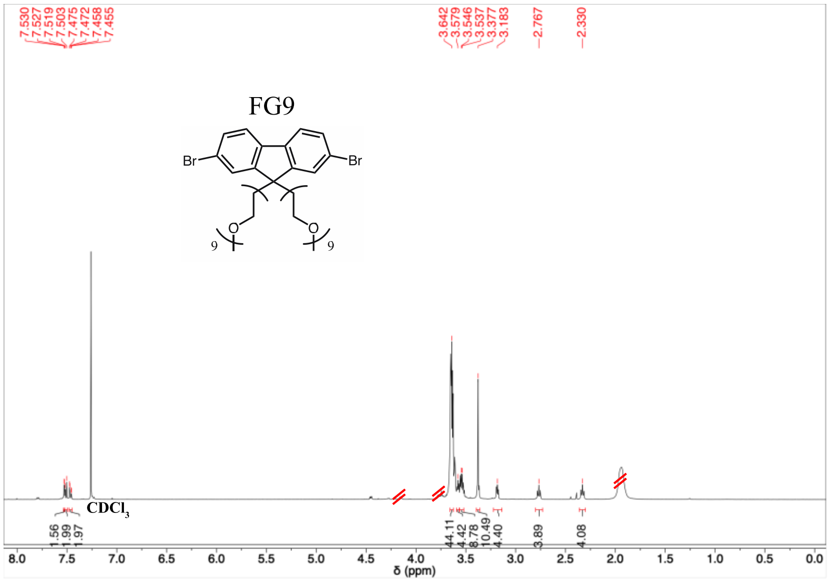

Figure S7: FG9 ^{1}H NMR with trace amount of THF and EtOAc.

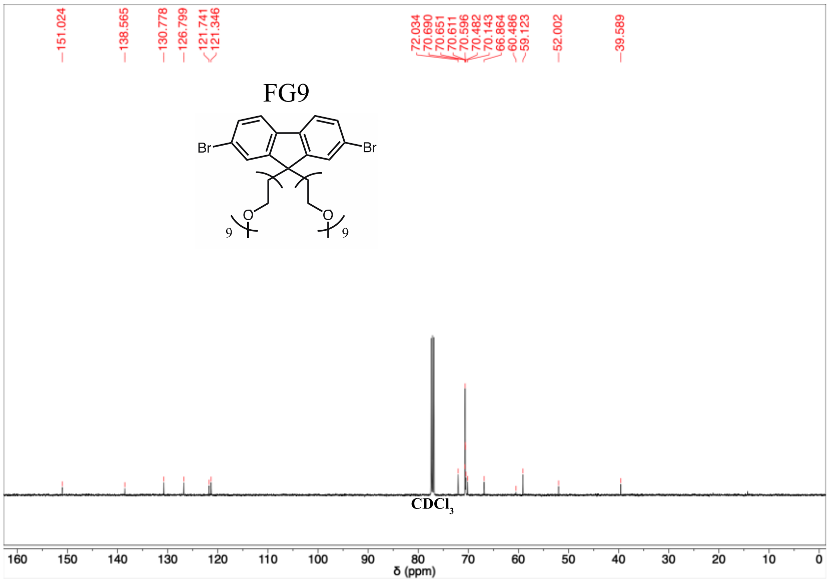

Figure S8: FG9 ^{13}C NMR

Synthesis of Neutral Polymers.

Polymer nPFNG9

Polymerization of poly([9,9-bis(3'-(N,N-dimethyl-amino)-propyl)-fluorene]-alt-co-[9,9-bis-(2-(2-(2-(2-(2-(2-(2-(2-(2-methoxy-ethoxy) ethoxy) ethoxy) ethoxy) ethoxy) ethoxy) ethoxy)

ethoxy) ethyl)- fluorene] To a clean and dried 48 mL pressure tube, a Teflon coated stir bar, FG9 (1140 mg, 1.00 mmol), FNB (580 mg, 0.99 mmol), potassium carbonate (K_2CO_3, 1.73 g, 12.5 mmol), 1,4- dioxane (dioxane, 10.0 mL, 136.0 mmol), DI H_2O (6.0 mL, 332.4 mmol), and Pd(dppf)Cl_2 (10 mg, 0.01 mmol) were added. The reaction solution as well as the head space was flushed with N_2(g). The pressure vial was capped quickly, placed into a silicon oil bath, stirred, and heated at 100 °C for 24 hours. To stop the reaction, the stirring function was turned off, the bottom water layer was removed, and an aliquot of nPFNG9 dispersed in dioxane was removed for further characterization. The polymer in dioxane (1 mL) was pipetted into DI H_2O (10 mL) to induce precipitation of nPFNG9. The water was decanted from the polymer and nPFNG9 was dried via vacuum filtration.[1,6] **Figure S9**: nPFNG9 ^{1}H NMR (800 MHz, $CDCl_3$): δ 7.78- 7.69 (br, 1.00 H), 3.62 (br, 3.72 H), 3.53 (br, 1.51 H), 3.42 (br, 0.35 H), 3.36 (br, 0.64 H), 3.26 (br, 0.43 H), 2.89 (br, 0.30 H), 2.56 (br, 0.32 H), 2.01 (br, 1.56 H), 0.96- 0.88 (br, 0.36 H) **Figure S10**: nPFNG9 ^{13}C NMR (800 MHz, $CDCl_3$): δ 207.1, 140.8, 127.4, 120.5, 72.1, 70.7, 70.2, 67.2, 60.1, 59.2, 45.5, 38.0, 31.1, 29.8, 22.3.

A Note on the Determination of the nPFNG9 Molecular Weight

Determination of the molecular weight for the neutral precursor nPFNG9 proved to be extremely challenging. This is because such long oEG sidechains lead the polymer to be highly amphiphilic and thereby render it insoluble in many common polar *and* nonpolar organic solvents used in size exclusion chromatography (SEC). We attempted to perform SEC measurements in three ways: in warm THF, DMF, and hot trichlorobenzene. We found nPFNG9 to be insufficiently soluble in all three solvents, rendering measurements unreliable. However, we also synthesized nPFNG6 – the analog of nPFNG9 with nonionic sidechains containing 6 ethyleneglycol units.

nPFNG6 was synthesized, purified, and isolated under identical conditions as nPFNG9. We were in fact able to perform SEC measurements for nPFNG6, obtaining a number-average molecular weight of 39,720 g/mol and a polydispersity of 1.16. This corresponds to a degree of polymerization of ~72 (36 repeat units). We believe that, had it been possible to measure the molecular weight of nPFNG9, a similar value as nPFNG9 would likely be obtained. This belief is supported by our very recent work (currently under review) where we made shorter polymers, which allowed us to directly compare the degree of polymerization for nPFNG6 and nPFNG9. We found that the two differed by ~8%.

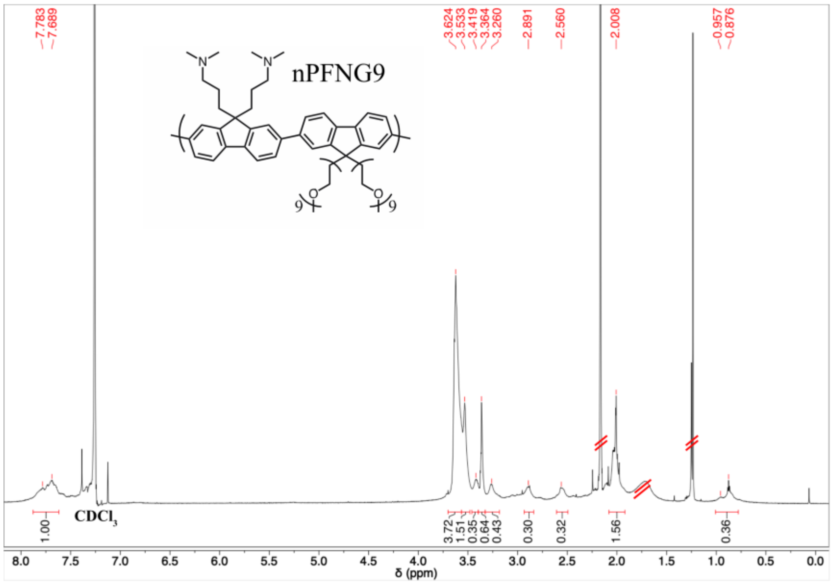

Figure S9: nPFNG9 ^{1}H NMR with trace amount of acetone, water, and pinacol.

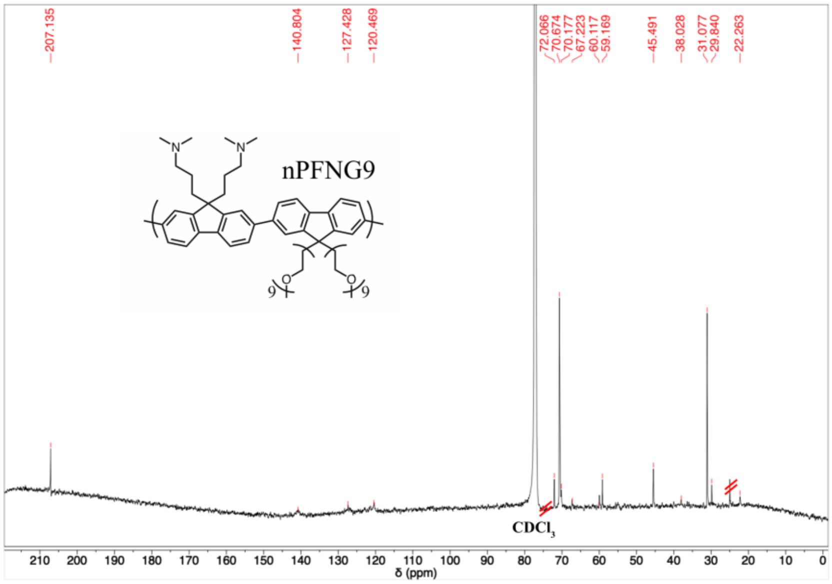

Figure S10: nPFNG9 ^{13}C NMR with trace amounts of pinacol.

Synthesis of Conjugated Polyelectrolytes.

Conjugated Polyelectrolyte PFNG9

Conjugated polyelectrolyte poly([9,9-bis(3’-(N,N,N-trimethyl-ammonium)-propyl)-fluorene]-alt-co-[9,9-bis-(2-(2-(2-(2-(2-(2-(2-(2-(2-methoxy-ethoxy) ethoxy) ethoxy) ethoxy) ethoxy) ethoxy) ethoxy) ethoxy) ethyl)- fluorene] The dioxane containing nPFNG9 was transferred into a 350 mL pressure vessel. Then quaternization occurred via the addition of methyl iodide (MeI, 1.0 mL, 16.1 mmol) directly to the remaining dioxane layer containing nPFNG9. The cap was threaded onto the pressure vessel, heated to 80 °C for 24 hours, and allowed to cool back down to room temperature before the cap was unscrewed. An aliquot of DI H_2O (200 mL) was added to help dissolve the precipitated polymer. The cap was threaded back onto the pressure vessel and was reheated to 80 °C for an additional 3 days. After which, the pressure vessel was cooled down to room temperature and uncapped. Once the cap was removed, the reaction contents were reheated to 80 °C to remove unreacted MeI from the reaction and reduced the volume of the reaction to 250

mL. The polymer solution was then dialyzed via a dialysis flask (10,000 MWCO) submerged in a vat of DI H_2O. The DI H_2O was replaced with fresh DI H_2O every day for 5 days. After 5 days, the dialyzed solution of PFNG9 was concentrated under reduced pressure, filtered, transferred into a 50 mL Falcon tube, and lyophilized to yield PFNG9 as a brown solid (320 mg, 24.6% yield).[1,6]

Figure S11: PFNG9 ^{1}H NMR (800 MHz, D_2O): δ 8.06- 7.93 (br, 1.00 H), 3.63 (br, 2.66 H), 3.58 (br, 2.27 H), 3.34 (br, 0.67 H), 3.28 (br, 0.17 H), 3.22 (br, 0.17 H), 2.98 (br, 0.17 H), 2.80 (br, 1.65 H), 2.68 (br, 0.06 H), 2.36 (br, 0.28 H), 1.17 (br, 0.38 H) **Figure S12**: PFNG9 ^{13}C NMR (800 MHz, D_2O): δ 151.2, 140.6, 139.8, 128.4, 127.1, 121.7, 120.8, 71.8, 71.0, 69.6, 67.1, 66.6, 66.3, 60.4, 58.1, 54.3, 52.6, 38.7, 35.2, 30.2, 17.7.

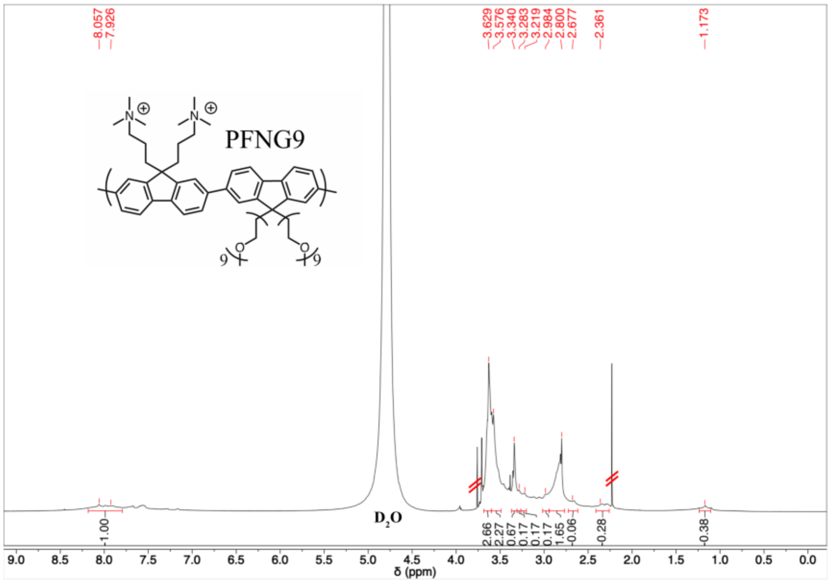

Figure S11: PFNG9 ^{1}H NMR with trace dioxane and acetone.

S2. Sample Preparation

Potassium bromide (KBr, 99.99 % purity) was obtained from Sigma-Aldrich. Tetraethylammonium bromide (> 98.0 % purity) (TEAB) from TCI Chemicals, and calcium

bromide ($CaBr_2$, extra pure) from Fisher Scientific. All chemicals were used as received. Stock solutions of 11 mg/mL PFNG9, 5.0 M KBr, 7.0 M LiBr, and 7.0 M TEAB were prepared using degassed (argon) HPLC grade water (Sigma-Aldrich). The PFNG9 stock was stirred at 70 °C for 6 hrs in a light protected vial. The salt stocks were stirred and heated at 70 °C for 15 min to guarantee all salt crystals were fully dissolved.

The PFNG9 concentration was fixed at 4.624 mg/mL for all solutions. Samples containing 5.0 M KBr were made with solid KBr; samples at 0.5 M and 2.5 M KBr were made using degassed KBr stock. The order of addition was as follows: KBr, HPLC water, PFNG9. Samples were stirred at 250 rpm at 70 °C for 6 hrs. Samples were allowed to cool to room temperature before any analysis was performed and care was taken to limit ambient light exposure. All other samples containing TEAB or $CaBr_2$ were made using degassed salt stocks in using the same prep described above.

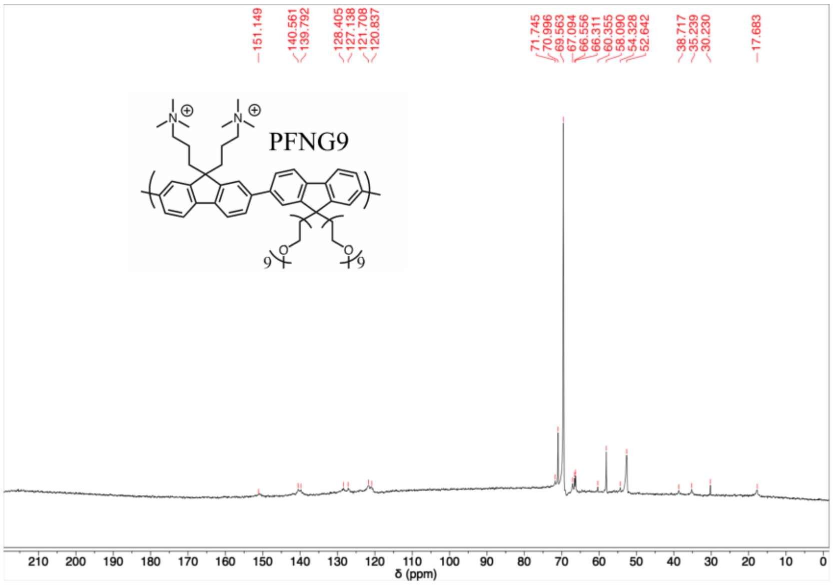

Figure S12: PFNG9 ^{13}C NMR

S3. Experimental Techniques

Microscopy. Images were acquired using a Leica DM5500 B widefield microscope equipped with a Leica DCF360 monochrome camera using ∞/0.17/o, HCX PL FLUORTAR 10x/0.3 and ∞/0.17/o, HCX PL FLUORTAR 40x/0.75 objectives. When needed, a plastic scoopula was used to dislodge the coacervate phase from the stir bar and sides of the vial. Samples were then mixed to homogenize the dilute and coacervate phases. Samples were excited using the following:

*Blue Channel (A4): excitation at 340-380 nm; emission at 450-490 nm

*Green Channel (GFP): excitation at 450-490 nm; emission at 500-550 nm

The A4 filter was chosen to select for the emission of the dilute phase and a GFP filter was used to select for the emission of the coacervate phase. Samples were also imaged using transmitted light DIC when using a 40x objective.

Five images of samples containing 2.5 M and 5.0 M KBr respectively were analyzed using the ImageJ-based software *Fiji.* ImageJ was used to identify the circumferences of coacervate droplets and measure their diameters (Figure SX).

A Leica SP5 Confocal Microscope was used to collect PL spectra from regions of interest in an image after performing a xyλ scan, in which the excitation wavelength was fixed, and the detected emission wavelength was scanned in 5-nm increments. Images were collected using a 20x/0.75 objective at 16-bit resolution. xyλ scans were taken while exciting with 405, 458, 476, and 496 nm laser lines, and emission was detected out to 750 nm.

Steady-State Photoluminescence Spectroscopy. Steady-state PL was collected using a home-built laser system described previously.[1] Samples were excited in a front-face geometry with 375-nm light from a pulsed picosecond diode laser (BDS-SM Series, Becker & Hickl GmbH),

and emission was collected between 400-700 nm using a PIXIS 100 CCD (Princeton Instruments).

Time-Resolved Photoluminescence Spectroscopy. Bulk-solution time-resolved photoluminescence (TRPL) measurements were collected using time-correlated single photon counting. Details of the home-built laser system and experimental setup have been described previously.[7] Samples were excited at 375 nm using a pulsed picosecond diode laser (BDS-SM Series, Becker & Hickl GmbH) or at 445 nm using a pulsed supercontinuum picosecond laser (Super K EXTREME, NKT Photonics) coupled to an acousto-optic filter and an external RF driver (Super K SELECT, NKT Photonics). The excitation beam was vertically polarized, and emission was collected in a front-face geometry with the emission polarizer set to the magic angle. PL lifetimes were determined using forward convolution with the measured instrument response function taken using a scattering Ludox sample. This was done using least-squares minimization via the DecayFit MATLAB package developed by Soren Preus (Fluorescence Decay Analysis Software 1.3, FluorTools, www.fluortools.com). A sum-of-exponentials model was used for the decay.

Fluorescence lifetime imaging (FLIM) measurements were carried out using a Zeiss LSM 980 NLO confocal microscope (Becker-Hickl TCSPC FLIM). Samples were excited using a 445-nm laser line, and PL was collected using a 590/50 nm filter cube. Images were collected using a 512 x 512 pixel resolution and a 50-s collection time. Fluorescence lifetime averages and distributions were determined using the SPCImage 8.5 NG software via the maximum likelihood estimation method.

Cryo-TEM. A sample droplet of 4 µL at 4.624 mg/mL PFNG9 and 5.0 M KBr was deposited on a C-flat holey carbon-coated TEM support grid (CF-2/2-2C from Electron Microscopy Services-

EMS) previously glow-discharged (PELCO easyGLOW) using 15 mA for 30 s. The sample was blotted for 2.5 s using a Vitrobot Mark IV (FEI Company) at 22 °C and ~100% humidity and sequentially fast-plunged into liquid ethane.

The images were aquired using 1-s exposure on a 4k x 4k CETA CCD Camera coupled to a ThermoFischer Glacios cryo-TEM operating at 200 kV. Images were collected at a nominal 2Å pixel size, 73,000 x magnification, and -3.5 µM defocus. Fiji – ImageJ was used for data analysis.

Size Exclusion Chromatography. Polymer molecular weight was measured by triple-detection size exclusion chromatography (SEC) using a Malvern OmniSEC equipped with refractive index, light scattering, and intrinsic viscosity detectors calibrated with a single poly(styrene) standard. Analysis was performed in tetrahydrofuran running at 1 mL min^{-1} and 35 ºC.

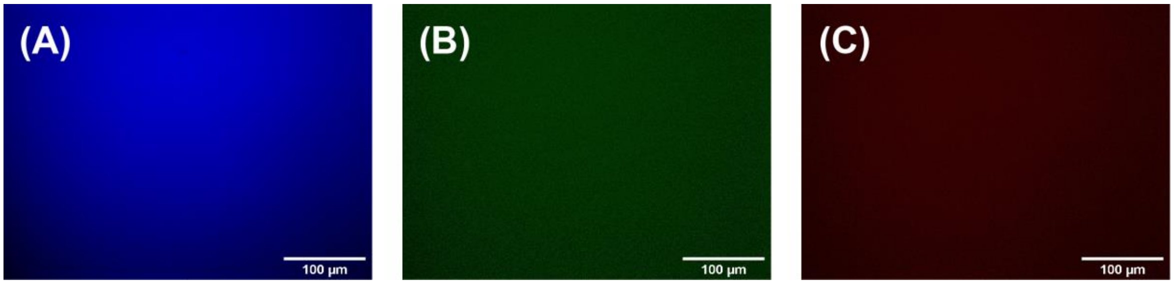

Figure S13: PL microscopy images of 4.624 mg/mL PFNG9 with three different filter settings. (A) Excitation between 340-380 nm and emission between 450-490 nm. (B) Excitation from 450-490 nm and emission between 500-550. (C) Excitation between 540-580 nm and emission between 540-608 nm.

S4. Supplemental Data

Optical Microscopy.

Figure S13 shows PL microscopy images of PFNG9 with no added salt at the fixed polymer concentration (4.624 mg/mL) used throughout this study. Images show fully dissolves

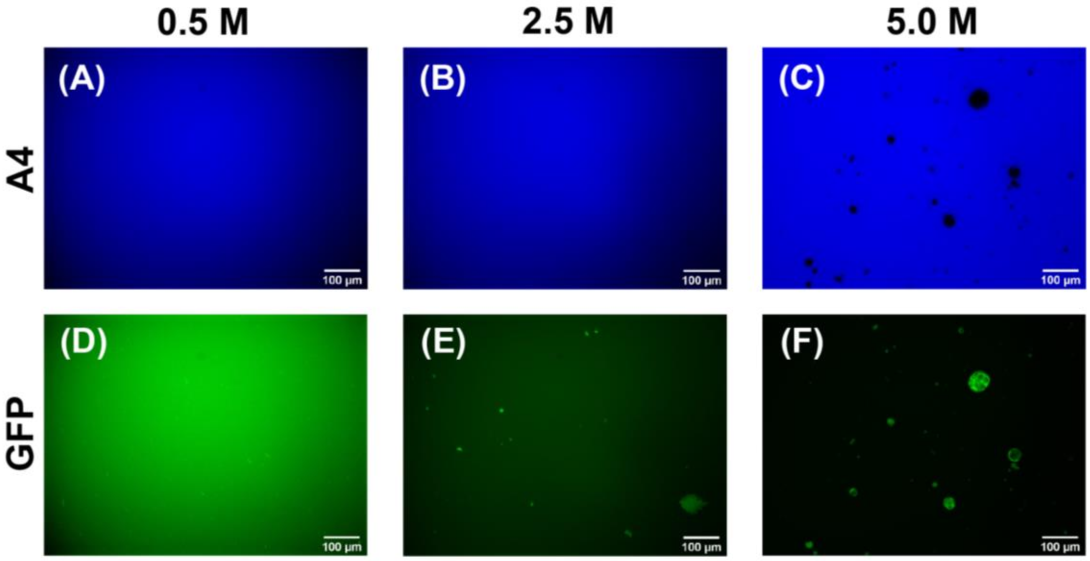

Figure S14: Fluorescence Microscopy images of PNFG9 with (A and D) 0.5 M KBr, (B and E) 2.5 M KBr, and (C and F) 5.0 M KBr using two different filters. Blue channel (A-C): Ex: 340-380 nm, Em:450-490 nm. Green channel (D-F) Ex: 450-490 nm, Em: 500-550 nm.

solutions and a strong fluorescence signal when using the A4 excitation and emission (Figure S13A) filter. Figure S14 shows the progression from fractal-like particles to the formation of droplets with increasing concentration of KBr. This is accompanied by a change in the photophysics where the particles at 2.5 M fluoresce within both the blue and green channels, but the droplets formed at 5 M show a darkening in the blue channel. Figures S15-S17 show microscopy images of PFNG9 with varying LiBr, tetraethylammonium bromide (TEAB), and $CaBr_2$. Figures show that PFNG9 remains fully dissolved regardless of LiBr or TEAB concentrations where the dark circular area in Figure S16A is simply a trapped air bubble between the microscope slide and coverslip evident by the lack of fluorescence. Figure S17

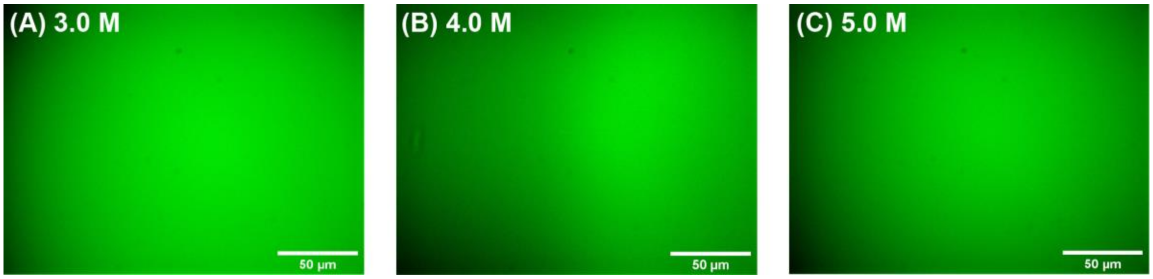

Figure S15: Fluorescence Microscopy images of PNFG9 with (A) 3.0 M LiBr, (B) 4.0 M LiBr, and (C) 5.0 M LiBr (Ex: 450-490 nm, Em: 500-550 nm).

shows that PFNG9 phase separates into fractal precipitant-like particulates when exposed to $CaBr_2$, with no evidence of droplet formation occurring at the representative concentrations. Additionally, these images represent PL (ex: 450-490 nm, em: 500-550 nm) that is distinctly different from the sample found to undergo coacervation (Figure S14C and S14F).

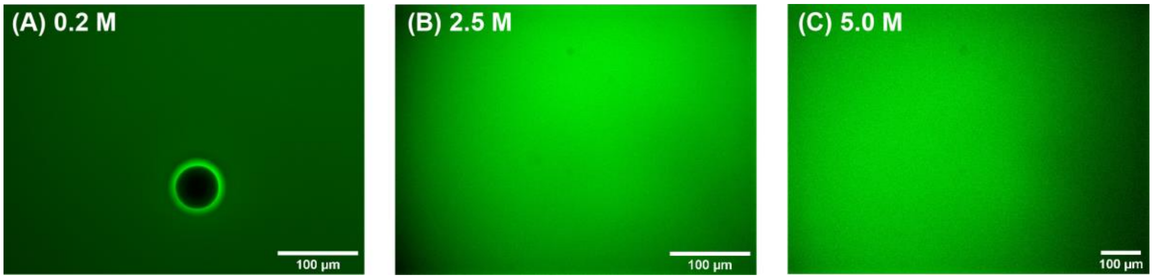

Figure S16: Fluorescence Microscopy images of PNFG9 with (A) 0.2 M TEAB, (B) 2.5 M TEAB, and (C) 5.0 M TEAB (Ex: 450-490 nm, Em: 500-550 nm) where the dark circular structure in panel A is a trapped air bubble.

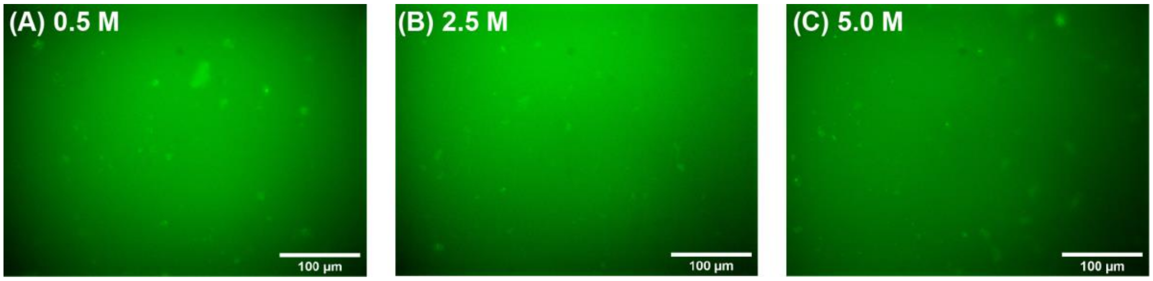

Figure S17: Fluorescence Microscopy images of PNFG9 with (A) 0.5 M $CaBr_2$, (B) 2.5 M $CaBr_2$, and (C) 5.0 M $CaBr_2$ (Ex: 450-490 nm, Em: 500-550 nm).

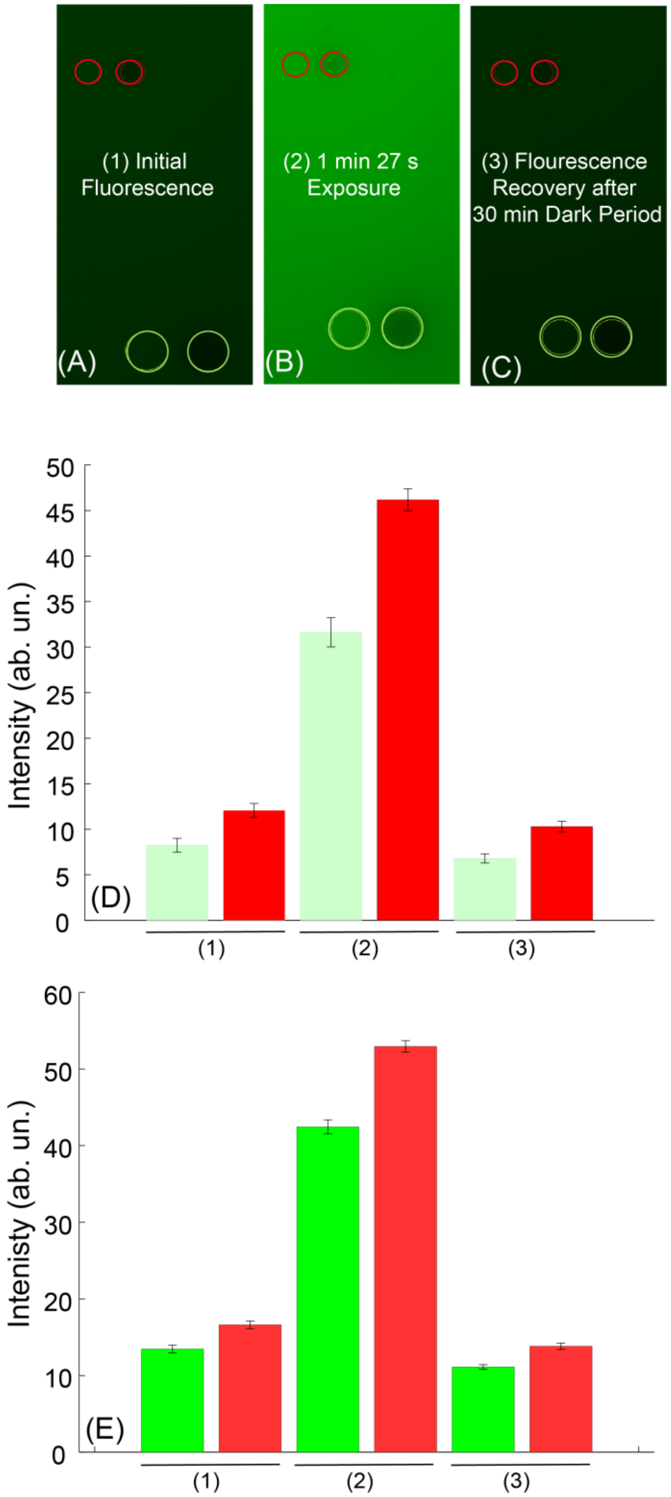

Figure S18: Fluorescence intensity recovery using excitation from 340-380 nm and emission from 450-490 nm.

Figures S18 and S19 show fluorescence recovery of the dilute solution and droplets by comparing original PL intensity, PL intensity after a ~90-s mercury lamp exposure, and PL intensity after a 30 min dark period.

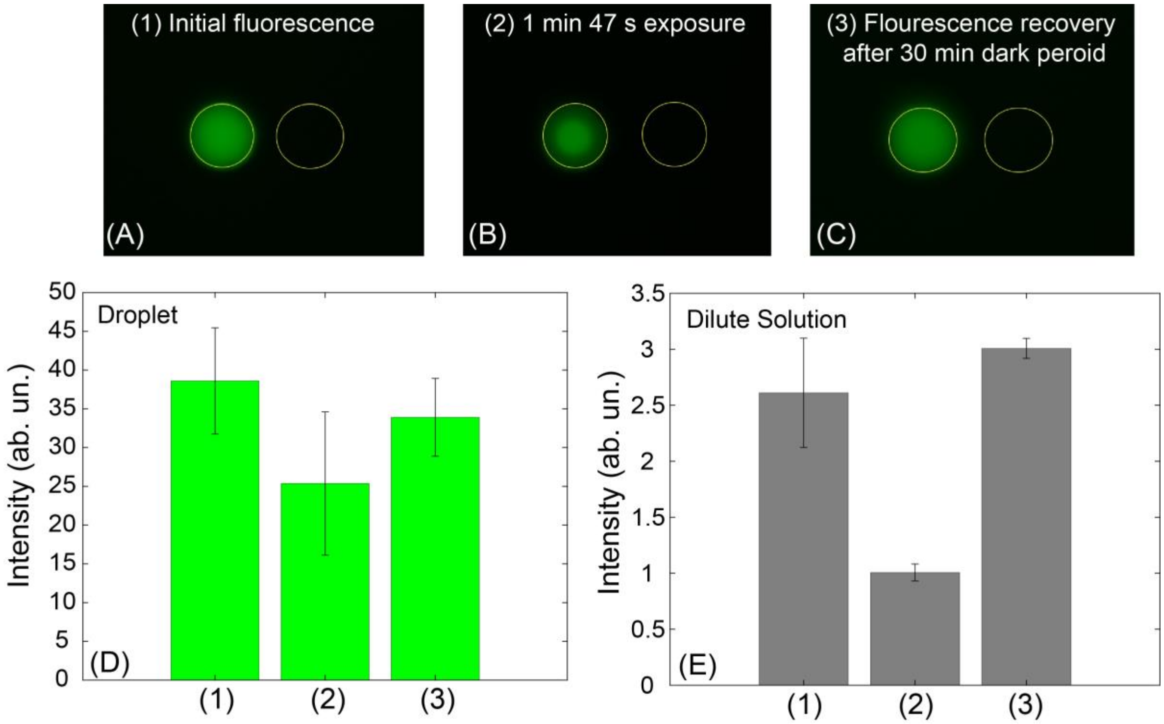

Figure S19: Fluorescence intensity recovery using excitation from 450-490 nm and emission from 500-550.

Steady-State Spectroscopy.

Figure S20 shows steady-state PL spectra of bulk PFNG9 solutions without added salt and with 0.5, 2.5, and 5.0 M KBr. Measurements were taken in triplicate. Dotted lines indicate averaged spectra. Figure S21A shows the averaged spectra plotted together and indicates PFNG9 sampled with KBr are all redshifted relative to the no-salt control. Panel S21B shows the dilute phase and the concentrated coacervate phase PL of the PFNG9 sample with 5.0 M KBr. The dilute phase was separated from the concentrated phase by ultracentrifugation. The concentrated phase was separated from the dilute phase by carefully scraping the viscous build-up from the

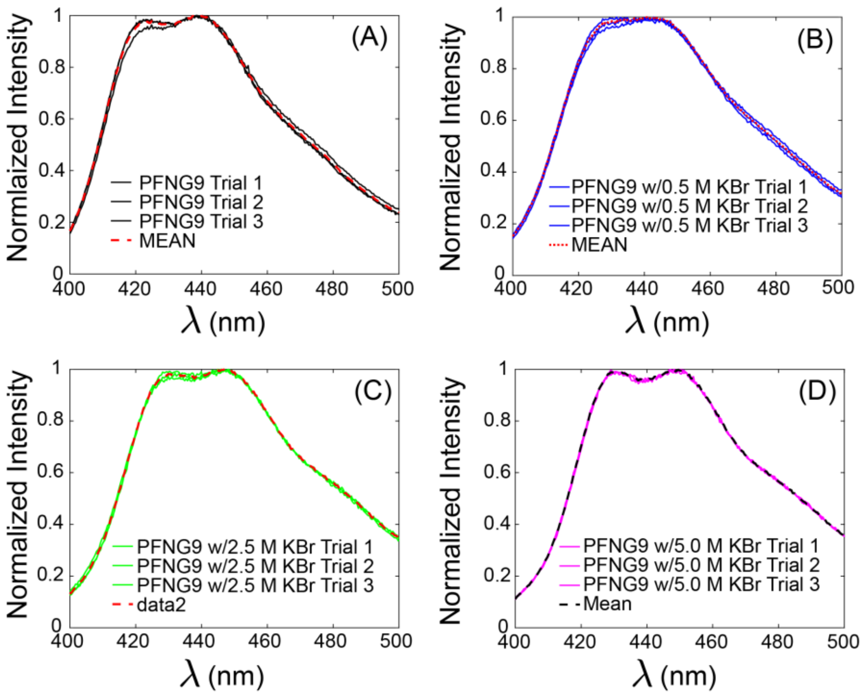

Figure S20: Three trials of steady-state PL of bulk solutions (i.e., dilute and concentrated phases together) are shown along with the averaged spectrum for (A) PFNG9, and PFNG9 with (B) 0.5 M KBr, (C) 2.5 M KBr, and (D) 5.0 M KBr.

sides of the glass vials and stir bar with a plastic scoopula and depositing it on a standard glass microscope slide, which was then covered with a coverslip and Kapton taped to minimize evaporation. PL was then taken by exciting the sample in a front-face geometry at a 45º angle at 375 nm. The concentrated coacervate phase was found to fluoresce more weakly compared to the dilute phase.

Figure S22 shows steady-state PL collected using an xyλ scan on a confocal microscope. PL signal was collected in triplicate and the average spectra are shown as black dotted lines. The averaged spectra are then compared in Figure S22D to highlight differences in PL signatures between the dilute solution and the concentrated phase. The droplet PL excitation wavelength dependance is shown in Figure S23 along with fits generated from one or two gaussians. Here, the gaussians capture the contribution from two distinct emitting states (i.e., components). and how the droplet PL is dependent on excitation wavelength.

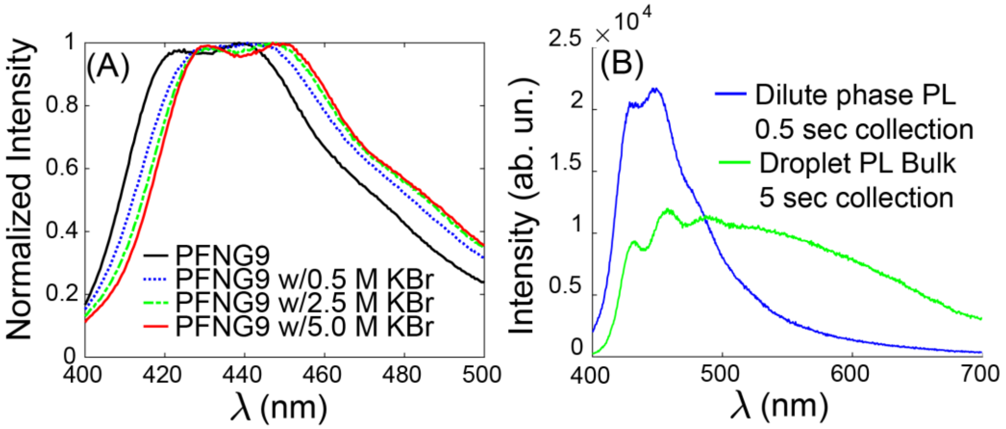

Figure S21: (A) Normalized steady-state PL comparison of bulk solutions of PFNG9 without added salt and with increasing concentration of KBr. (B) PL intensity comparison between the concentrated phase of PFNG9 with 5.0 M KBr and the dilute phase. The collection times refer to how long the detector was allowed to receive emitted light from the sample during the experiment.

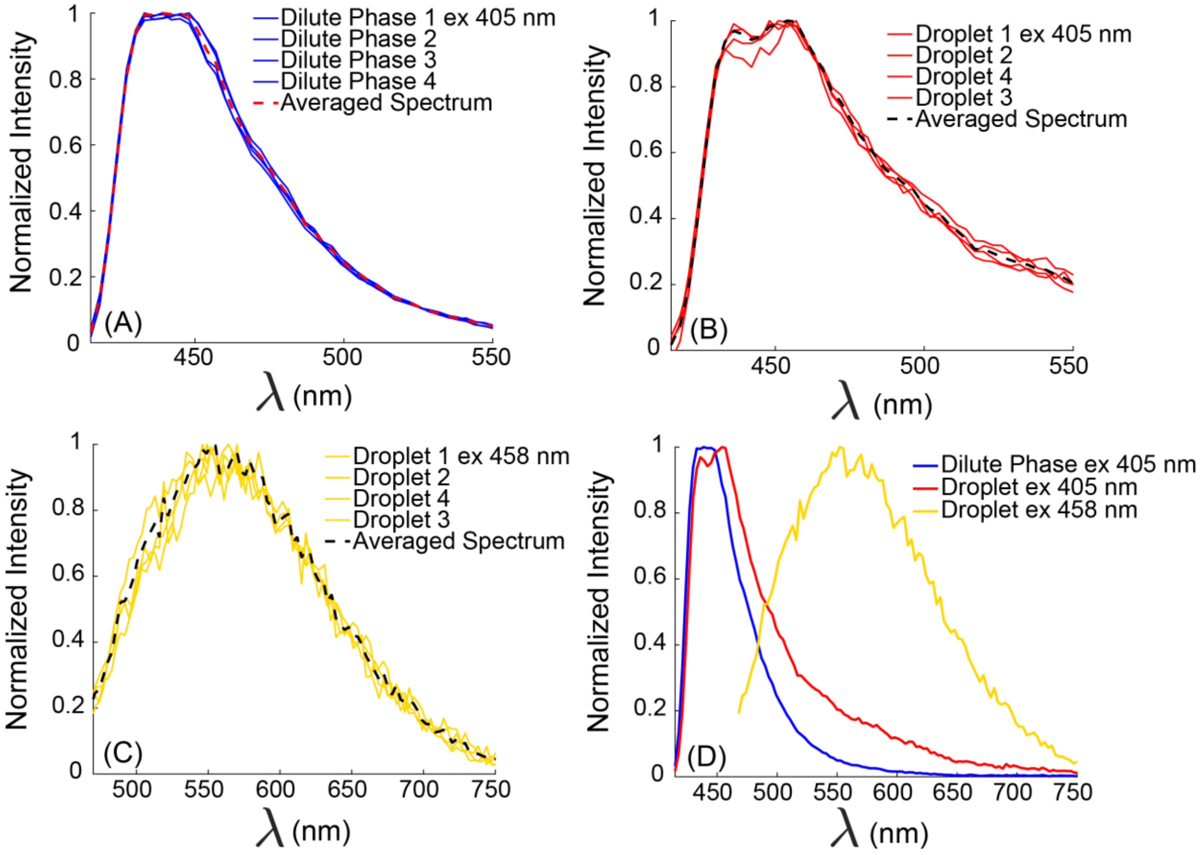

Figure S22: Normalized triplicate PL measurements collected using confocal xyλ scans shown with averaged spectra for (A) the dilute phase excited at 405 nm, (B) droplets excited at 405 nm and (C) 458 nm. (D) shows averaged PL spectra of dilute phase and droplets plotted together.

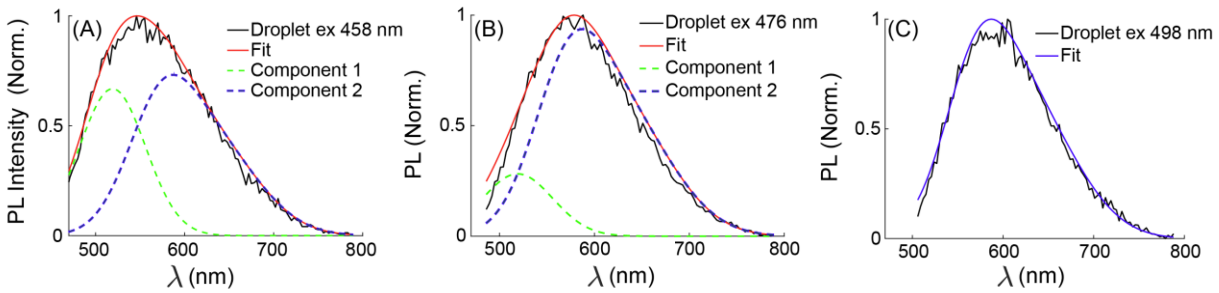

Figure S23: Fitting of droplet PL spectra from confocal images following excitation at (A) 458 nm, (B) 476 nm, and (C) 498 nm. The spectrum after excitation at 498 nm (C) was fit to a sum of two Gaussians. The addition of the second Gaussian significantly improved the fit quality. The spectrum under excitation with 458-nm light (A) was fit to a linear combination of one variable Gaussian on the blue side and the rigidly fixed two-Gaussian fit from (C), with only its relative amplitude allowed to vary. The spectrum in (B) was then fit to a linear combination of the two contributions in (A) but with variable coefficients but with all other parameters (peak positions, widths) held fixed.

Cryo-TEM.

Figure S24A shows a cryo-TEM image of a coacervate droplet. The dark regions correspond to fluctuations in local KBr concentration near the onset of crystallization. The latter was confirmed by the presence of weak Bragg reflections at large scattering angles. Figure S24B shows another image in which multiple droplets overlap with one another.

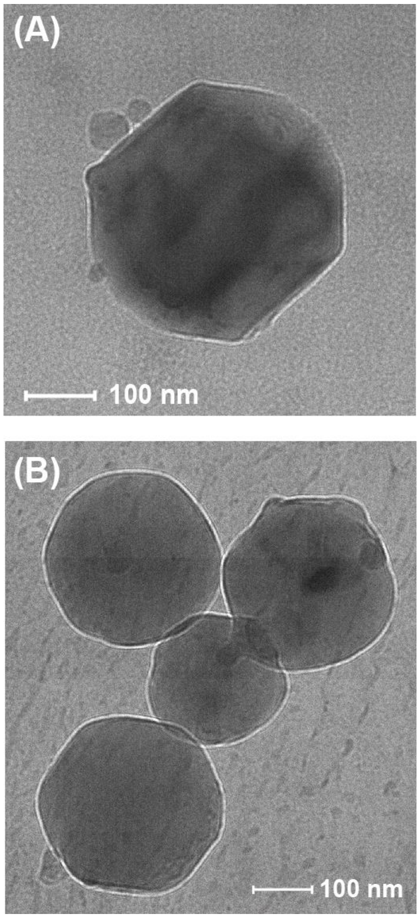

Figure S24: Cryo-TEM image of coacervate droplets exhibiting inhomogeneous contrast regions within the droplet interior (A). (B) shows an image of overlapping, mesoscale coacervate droplets.

Time-Resolved Photoluminescence Spectroscopy.

Figure S25 shows TRPL decays for bulk solutions of PFNG9 with no added salt and with 0.5, 2.5, and 5.0 M KBr excited at 375 nm; emission was collected at 420 nm. Figure S25A shows a slight increase in PL lifetime with increasing KBr. The short component of each lifetime and

the average lifetime for each [salt] are plotted in panel S25B. Figure S26 shows TRPL decays for the separated concentrated phase as described above. Samples were excited at 375 nm, and emission was collected at 420, 460, 500, and 600 nm respectively. A gradual increase in average PL lifetime is seen when collecting at redder emission wavelengths. The largest average lifetime was 1.34 ns.

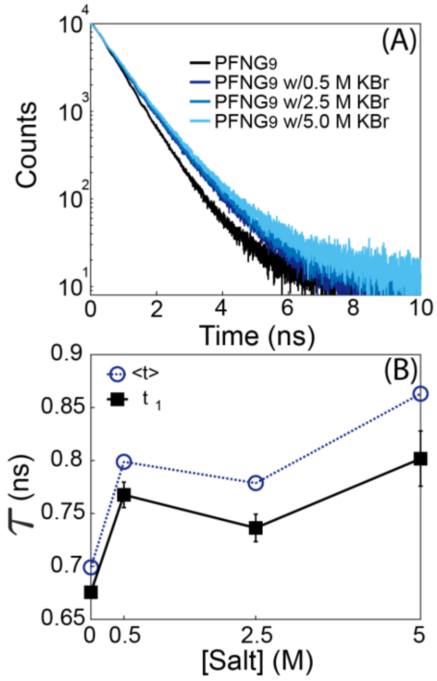

Figure S25: (A)TRPL decays found for the bulk solution of PFNG9 with increasing concentrations of KBr. (B) shows the faster component of the bi-exponential decay lifetime plotted along with $\langle\tau\rangle$.

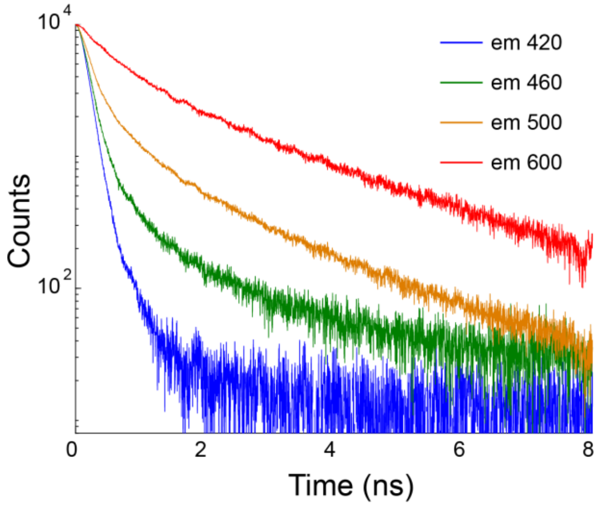

Sample	a_1	τ_1 (ns)	a_2	τ_2 (ns)	$\langle\tau\rangle$ (ns)
PFNG9 w/5.0 M KBr CP Em 420 nm	1	0.12	-	-	0.12
Em 460 nm	0.998	0.36	0.002	3.1	0.41
Em 500 nm	0.980	0.55	0.020	3.1	0.81
Em 600 nm	0.924	0.69	0.076	3.1	1.34

Figure S26: TRPL decays of the concentrated phase of PFNG9 with 5.0 M KBr where excitation was fixed at 375 nm and the wavelength of emission collected was varied.

Figure S27 shows FLIM images and PL lifetime distributions for 9 droplets excited at 445 nm and emission collected at 590/50 nm.

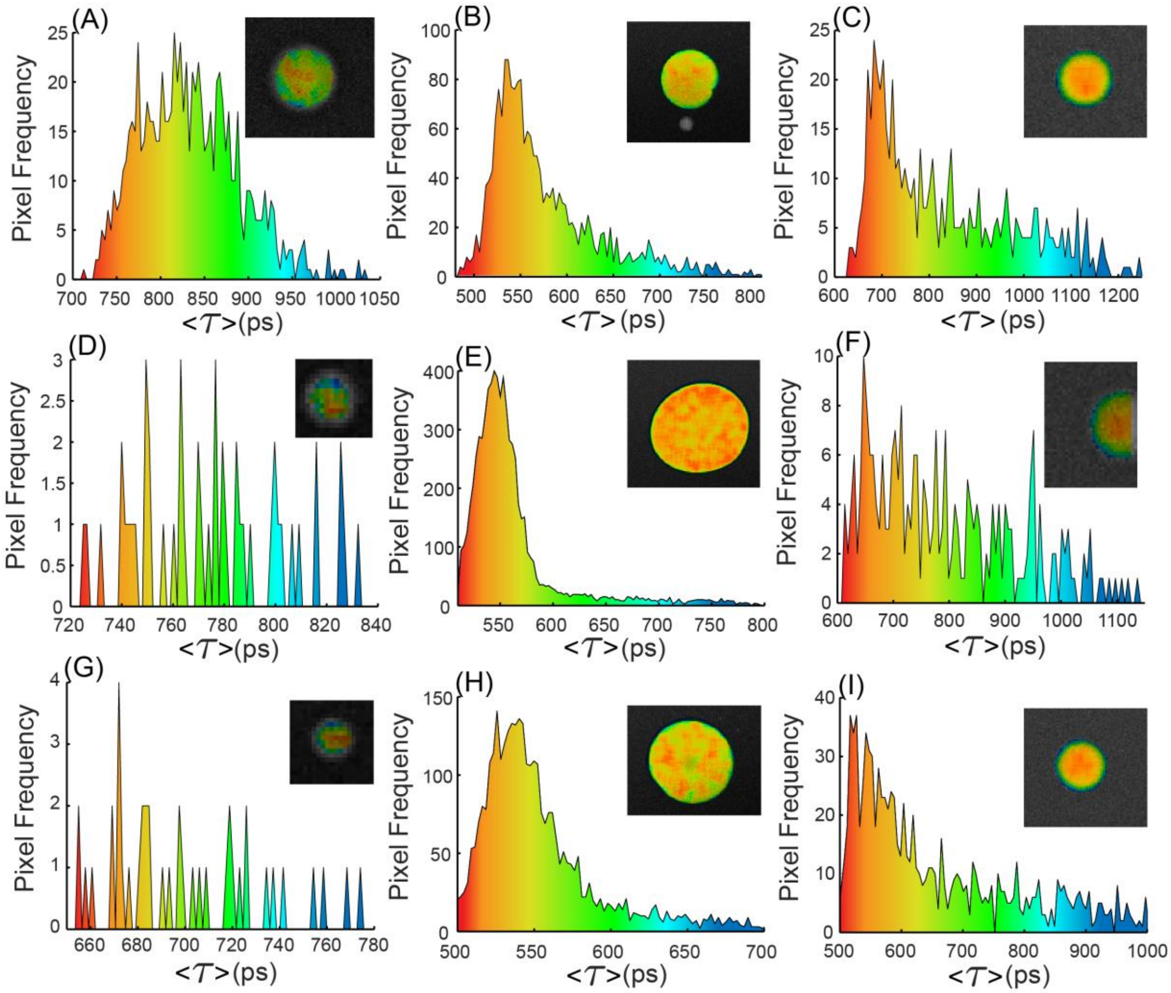

Figure S27: FLIM lifetime distributions from 9 different coacervate droplets where the color coding of the PL lifetime histogram matches the colors displayed in the images. Lifetime heat maps for the corresponding droplets are shown as insets.

Additional Wide-field Fluorescence Microscopy.

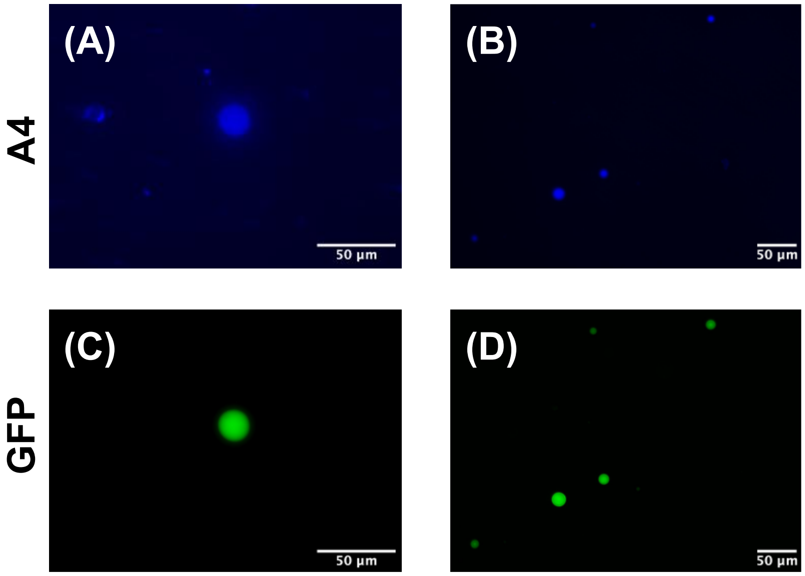

Figure S28: Wide-filed fluorescence microscopy images of PFNG9 with 5M KF using two different filters, showing formation of simple coacervate droplets. Blue channel (A and B) Ex: 340-380 nm, Em: 450-490 nm. Green channel (C and D) Ex: 450-490 nm, Em: 500-550 nm.

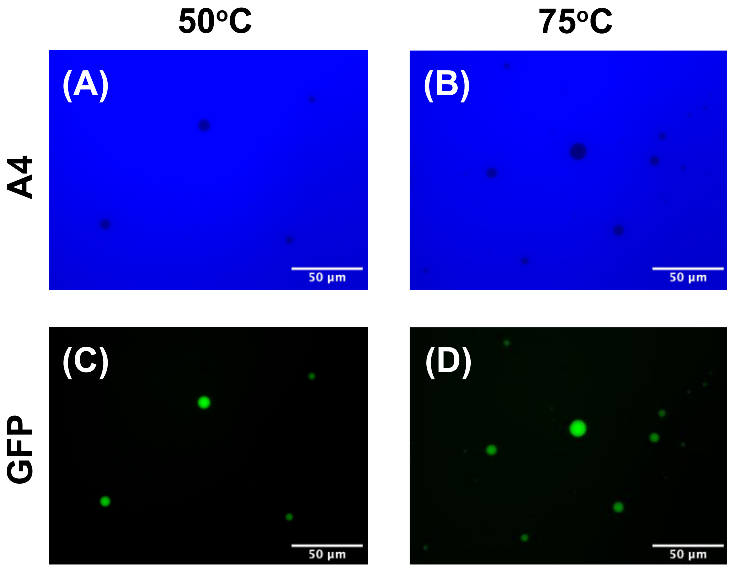

Figure S29: Wide-filed fluorescence microscopy images of PFNG9 samples with 5M KBr heated at 50 °C for one hour and cooled to room temperature (A and C), and samples heated to 75 °C for an hour before cooling to room temperature (B and D) using two different filters. Blue channel (A and B) Ex: 340-380 nm, Em: 450-490 nm. Green channel (C and D) Ex: 450-490 nm, Em: 500-550 nm.

Movie S1: Slow droplet dynamics. The top middle of the field of view shows the gradual thinning of a neck connecting two adjacent droplets.

References

(1) Pu, K.-Y.; Wang, G.; Liu, B. Design and Synthesis of Conjugated Polyelectrolytes; John Wiley & Sons Inc., New Jersey, **2013**.

(2) Heathcote, R.; Howell, J. A. S.; Jennings, N.; Cartlidge, D.; Cobden, L.; Coles, S.; Hursthouse, M. Gold(i)–Isocyanide and Gold(i)–Carbene Complexes as Substrates for the Laser Decoration of Gold onto Ceramic Surfaces. *Journ. Chem. Soc. Dalton T,* **2007**, *12*, 1309–1315.

(3) Meng, B.; Song, H.; Chen, X.; Xie, Z.; Liu, J.; Wang, L. Replacing Alkyl with Oligo(Ethylene Glycol) as Side Chains of Conjugated Polymers for Close π–π Stacking. *Macromolecules*, **2015**, *48*, 4357–4363.

(4) Wang, H.; Lu, P.; Wang, B.; Qiu, S.; Liu, M.; Hanif, M.; Cheng, G.; Liu, S.; Ma, Y. A Water-Soluble Π-Conjugated Polymer with up to 100 Mg · ML−1 Solubility. *Macromol Rapid Comm*, **2007**, *28*, 1645–1650.

(5) Huang, F.; Wu, H.; Wang, D.; Yang, W.; Cao, Y. Novel Electroluminescent Conjugated Polyelectrolytes Based on Polyfluorene. *Chem Mater.* **2004**, *16*, 708–716.

(6) Pu, K.; Liu, B. Conjugated Polyelectrolytes as Light-Up Macromolecular Probes for Heparin Sensing. *Adv Funct Mater* **2009**, *19*, 277–284.

(7) Johnston, A. R.; Perry, S. L.; Ayzner, A. L. Associative Phase Separation of Aqueous π-Conjugated Polyelectrolytes Couples Photophysical and Mechanical Properties. *Chem. Mater.* **2021**, *33*, 1116–1129.
