# Supplementary material for: Excitonically Coupled Simple Coacervates via Liquid/Liquid Phase Separation
Source: J Phys Chem Lett. 2022 Oct 28;13(44):10275–81. doi: 10.1021/acs.jpclett.2c02466 (PMC9661528; doi:10.1021/acs.jpclett.2c02466)
Supplement: Supplementary file 3 — jz2c02466_si_003.pdf [file jz2c02466_si_003.pdf]

jz-2022-02466c.R1

Name: Peer Review Information for "Excitonically Coupled Simple Coacervates via Liquid/Liquid Phase Separation"

First Round of Reviewer Comments

Reviewer: 1

Comments to the Author

What is the major advance reported in the paper?

This paper provides the first demonstration of liquid/liquid phase separation in conjugated polyelectrolytes in which the coacervate is demonstrated to be excitonically active. I think this is an outstanding result that is worthy of publication in JPC Letters.

What is the immediate significance of this advance?

This result conceivably opens up a new field in which the optoelectronic properties of coacervates can be tuned along with their thermodynamic and mechanical properties, potentially leading to some exciting multifunctional soft materials. The result also provides additional evidence addressing the controversial g-band of polyfluorenes. The FLIM analysis of the photophysics as a function of distance from the interface is interesting and can potentially provide a means of dissecting the photophysics of conjugated polymer interfaces in the future.

Technical suggestions?

The fact that a single conjugated polyelectrolyte species forms a coacervate is not unprecedented (e.g. “self-coacervates” in block polyampholytes (PNAS 2019 116 (17) 8224-8232.). However, the fact that the formation is so dependent on the identity of the cation is strange, and I, like the authors, don’t understand the thermodynamic driving force. I suppose that the fact that KBr is better absorbed compared to other salts means that there is better electrostatic screening within the coacervate – moreover, I can imagine that this might hinder orientations of stiff (hydrophobic) polyfluorene chains and hence reduce the propensity to stacking and aggregation. Regardless, the result itself is novel and exciting enough that I think these details can be reserved for an in depth follow up manuscript trying to understand the mechanisms of formation.

One point I might suggest to the authors - my intuition is that divalent Ca2+ should be better absorbed than even K1+. This is obviously proven wrong by their testing other salts. To further test the validity of the strong abdsorption of potassium, could the authors test another salt that also includes potassium?

How temperature stable are these droplets? I could not find any information on their existence at other temperatures. If the coacervation is driven by potassium ions, I am inclined to think they should exhibit

strong temperature dependence and that one could manipulate the necessary salt concentrations along with the droplet temperature to derive a minimal coexistence curve.

Can the authors provide any information on the chain density within the coacervate droplets relative to the supernatant?

Can the radial electronic gradient observed in the coacervate droplet FLIM analysis be used to demonstrate any kind of radial exciton transport within the droplet? If one could selectively segregate catalytic sites to the droplet interface then this could be an interesting way to do photodriven catalysis. One assumes that if the coacervate droplets can be made smaller and smaller, the radial gradient in optical properties should become larger and more dramatic.

Page 12 – I doubt these CPE chains are long enough to be entangled in the polymer dynamics sense of the word, so I would suggest the authors do not use this term. On this matter – how many repeat units are the average CPE chains?

For what it’s worth, the recent review by Rumyantsev and de Pablo (Annu. Rev. Condens. Matter Phys 12 (1), 155-176) explicitly discusses semiconducting coacervates and their emerging need, so it might be a more relevant generic review article to the authors’ interests than the Sing review.

Author's Response to Peer Review Comments:

Dear Prof. Editor,

Thank you very much for allowing us to revise our original submission. We believe we were able to satisfactorily address all reviewer comments. This led us to perform several additional experiments, which we believe have strengthened our original conclusions and improved the quality of the manuscript. Our responses and changes are detailed in the point-by-point response document. Changes made in the manuscript and the Supporting Information have been labeled with red text and submitted as materials for review.

As mentioned at the original submission stage, we are happy to participate in the transparent review process.

Thank you for your further consideration.

Sincerely,

Alex Ayzner

RESPONSE TO REVIEWERS

Reviewer queries are indicted by blue text following Q. Our responses are in black following R.

Q1: One point I might suggest to the authors - my intuition is that divalent Ca2+ should be better absorbed than even K1+. This is obviously proven wrong by their testing other salts. To further test the validity of the strong abdsorption of potassium, could the authors test another salt that also includes potassium?

R1: The reviewer's intuition matched ours, and we were similarly surprised. The suggestion to test another potassium salt is quite welcome. To address this question, we chose KF as a simple comparison to KBr but one that would likely reveal differences in phase behavior due to the anion identity if this were a prominent effect.

We find that at 5 M KF, the droplets persist, stregnthening the hypothesis that the K^+ - oEG interaction is implicated in the formation of the PFNG9 simple coacervate. The corresponding images are shown in the Supporting Information as new Figure S28.

The following text has been added on p. 13 of the revised manuscript:

"We also investigated whether the choice of anion played an important role in the phase behavior. We chose to evaluate whether coacervate formation took place in the presence of 5 M KF, with the expectation that the fluoride anion was substantially different in size and charge density compared to the bromide anion. We found that coacervate droplets were formed with KF as with KBr (see Figure S28)."

Q2: How temperature stable are these droplets? I could not find any information on their existence at other temperatures. If the coacervation is driven by potassium ions, I am inclined to think they should exhibit strong temperature dependence and that one could manipulate the necessary salt concentrations along with the droplet temperature to derive a minimal coexistence curve.

R2: To help answer this question, we heated the phase-separated solution with 5 M KBr first to 50 ºC for 1 hour. Then the same solution was then heated to 75 ºC for another hour. Visual inspection showed that the amount of concentrated visibly phase decreased at 75 ºC, suggesting increased proximity to the binodal in thermodynamic state space. When the phase mixture was cooled back down to room temperature from either elevated temperature, coacervate droplets were again observed under the microscope, as shown in the Supporting Information in the new Figure S29. This suggests that the simple coacervate phase is very likely the global equilibrium state of this system at room temperature.

The following sentence been added on p. 14 of the revised manuscript:

"Nevertheless, the coexistence of a dilute solution with a simple coacervate phase appears to correspond to a global free energy minimum at room temperature (Figure S29)."

Separately, we have also been in the process of setting up temperature-dependent PL microscopy measurements. This setup is currently being tested prior to conducting accurate measurements. These measurements will contribute to a detailed follow-on investigation of the phase behavior as a function of polyelectrolyte concentration, oEG chain length and over an extended salt concentration range.

Q3: Can the authors provide any information on the chain density within the coacervate droplets relative to the supernatant?

R3: The highly viscous and adhesive nature of the simple coacervate phase has so far prevented us from performing accurate volume measurements of the concentrated phase, which could then be used together with absorption measurements to calculate the polyelectrolyte concentration in both the dilute and concentrated phases. We were able to do so previously when the concentrated phase was a colloidal gel or a complex fluid. In the follow-on work that is currently in progress, we are taking advantage of a significant increase in the amount of the coacervate phase formed at higher CPE concentrations to increase the probability that the volume measurement is accurate. We hope that this will allow us to perform accurate measurements of CPE concentration within the simple coacervate phase following ultracentrifugation.

Q4: Can the radial electronic gradient observed in the coacervate droplet FLIM analysis be used to demonstrate any kind of radial exciton transport within the droplet? If one could selectively segregate catalytic sites to the droplet interface then this could be an interesting way to do photodriven catalysis. One assumes that if the coacervate droplets can be made smaller and smaller, the radial gradient in optical properties should become larger and more dramatic.

R4: We appreciate the reviewer's fascinating idea. Our FLIM measurements focused on relatively large droplets to simplify the measurement by minimizing the droplet diffusion rate. The diffusion of small droplets made confocal measurements substantially more challenging. Nevertheless, the suggestion to probe smaller droplets and evaluate whether the radial lifetime gradient becomes more pronounced is quite intriguing. The idea of selectively segregating catalytic sites at the surface (if possible) is enticing.

Demonstrationg of radial exciton transport would require forming a highly localized exciton population and following its radial spread from few to 100s of ps. Although such an experiment is in principle possible, doing so in practice is quite involved. We believe such a measurement falls outside the scope of the current investigation.

Q5: Page 12 – I doubt these CPE chains are long enough to be entangled in the polymer dynamics sense of the word, so I would suggest the authors do not use this term. On this matter – how many repeat units are the average CPE chains?

R5: To address the reviewer's concern, we have changed "entangled" to "strongly interacting", which more closely reflects what we originally had in mind. We estimate that the degree of polymerization is approximately 70 (in fluorene units). This information has now been added to the Supporting Information.

Q6: For what it's worth, the recent review by Rumyantsev and de Pablo (Annu. Rev. Condens. Matter Phys 12 (1), 155-176) explicitly discusses semiconducting coacervates and their emerging need, so it might be a more relevant generic review article to the authors' interests than the Sing review.

R6: We thank the reviewer for this suggestion. This reference has now been incorporated.
